# Avatar-based patient monitoring improves information transfer, diagnostic confidence and reduces perceived workload in intensive care units: computer-based, multicentre comparison study

**DOI:** 10.1038/s41598-023-33027-z

**Published:** 2023-04-11

**Authors:** Lisa Bergauer, Julia Braun, Tadzio Raoul Roche, Patrick Meybohm, Sebastian Hottenrott, Kai Zacharowski, Florian Jürgen Raimann, Eva Rivas, Manuel López-Baamonde, Michael Thomas Ganter, Christoph Beat Nöthiger, Donat R. Spahn, David Werner Tscholl, Samira Akbas

**Affiliations:** 1 Institute of Anaesthesiology, University of Zurich and University Hospital Zurich, Zurich, Switzerland; 2 Department of Epidemiology and Biostatistics, University of Zurich, Zurich, Switzerland; 3 Department of Anaesthesiology, Intensive Care, Emergency and Pain Medicine, University Hospital Wuerzburg, University of Wuerzburg, Wuerzburg, Germany; 4 Department of Anaesthesiology, Intensive Care Medicine and Pain Therapy, University Hospital Frankfurt, Goethe University Frankfurt, Frankfurt, Germany; 5 Department of Anaesthesiology, Intensive Care Medicine and Pain Therapy, Hospital Clinic of Barcelona, University of Barcelona, Barcelona, Spain; 6 Institute of Anaesthesiology and Critical Care Medicine, Clinic Hirslanden Zurich, Zurich, Switzerland

**Keywords:** Health care, Medical research, Information technology

## Abstract

Patient monitoring is the foundation of intensive care medicine. High workload and information overload can impair situation awareness of staff, thus leading to loss of important information about patients’ conditions. To facilitate mental processing of patient monitoring data, we developed the Visual-Patient-avatar Intensive Care Unit (ICU), a virtual patient model animated from vital signs and patient installation data. It incorporates user-centred design principles to foster situation awareness. This study investigated the avatar’s effects on information transfer measured by performance, diagnostic confidence and perceived workload. This computer-based study compared Visual-Patient-avatar ICU and conventional monitor modality for the first time. We recruited 25 nurses and 25 physicians from five centres. The participants completed an equal number of scenarios in both modalities. Information transfer, as the primary outcome, was defined as correctly assessing vital signs and installations. Secondary outcomes included diagnostic confidence and perceived workload. For analysis, we used mixed models and matched odds ratios. Comparing 250 within-subject cases revealed that Visual-Patient-avatar ICU led to a higher rate of correctly assessed vital signs and installations [rate ratio (RR) 1.25; 95% CI 1.19–1.31; P < 0.001], strengthened diagnostic confidence [odds ratio (OR) 3.32; 95% CI 2.15–5.11, P < 0.001] and lowered perceived workload (coefficient − 7.62; 95% CI − 9.17 to − 6.07; P < 0.001) than conventional modality. Using Visual-Patient-avatar ICU, participants retrieved more information with higher diagnostic confidence and lower perceived workload compared to the current industry standard monitor.

## Introduction

Working in an intensive care unit (ICU) is demanding as it includes responsibility for several patients, management of emergencies and tracking a variety of monitoring and laboratory parameters^1,2^. Clinical severity scores indicate an increasingly sick patient population for whom medical advances provide growing monitoring options and therapies^3,4^. Therefore, it is not surprising that ICUs are among the most technologically enhanced medical areas to manage severely ill patients with complex medical needs^5^.

Situation awareness is a psychological concept pioneered by Mica Endsley, including the three levels of data perception, comprehension and anticipation of future states from the surrounding environment. It is a prerequisite for appropriate decision-making^6,7^. Demanding working conditions with high information load can lead to deterioration of situation awareness and thus expose patients to medical errors^1,8–10^. In contrast, improving the working environment according to user-centred design principles may help healthcare providers cope with overload and increase situation awareness^11–14^. Policymakers like the U.S. Food and Drug Administration and the International Electrotechnical Commission have identified that facilitating interaction between users and medical devices is essential for safety^15,16^. With few exceptions, there have yet to be any breakthrough innovations in presenting monitoring data in everyday intensive care practice in the last decades, although there is a demand for implementing new monitor technologies^8,12,17–20^. The Visual-Patient-avatar, a virtual patient model animated from alphanumerical monitoring data, has been developed as a new monitor modality aimed at fostering situation awareness. Various computer-based, eye tracking and high-fidelity simulation studies have validated the concept of vital sign monitoring primarily in a simulated anaesthesia context^21,22^. With the first version of Visual-Patient-avatar approaching its clinical release, extensions were made to adapt it to critical care requirements and offer the potential advantages of this visualisation technique in intensive care medicine. Compared to the Visual-Patient-avatar base version, Visual-Patient-avatar ICU newly displays patient installations, e.g., vascular lines, airway devices, sensors and additional vital signs such as the cardiac index or peak inspiratory pressure.

This study aims to evaluate for the first time the impact of the extended Visual-Patient-avatar ICU technique on information transfer, diagnostic confidence and perceived workload compared to conventional monitor modality. To determine the hypothesised superiority of this novel data presentation method, we conducted a multicentre computer-based study involving nurses and physicians working on different patient scenarios with either Visual-Patient-avatar ICU or a conventional monitor.

## Methods

The Cantonal Ethics Committee of Zurich, Switzerland, reviewed the study protocol and issued a declaration of no objection (Business Management System for Ethics Committees Number Req-Req-2021-00102). The study was performed in accordance with the Declaration of Helsinki and the International Ethical Guidelines for Biomedical Research involving human subjects. Nevertheless, we collected all data under written informed consent from all participants.

### Visual-Patient-avatar technique

The Visual-Patient-avatar technique has been developed and studied over the last ten years by our research group at the University Hospital Zurich, Switzerland. The technique pre-processes the monitoring data of actual patients and presents it in a visualised form: an avatar. This innovative monitor modality strongly incorporates user-centred design principles in order to improve situation awareness. Various numerical and waveform vital signs are synthesised and simplified into a single indicator that allows parallel information transfer and peripheral vision monitoring^21,23,24^.

**Figure 1:**
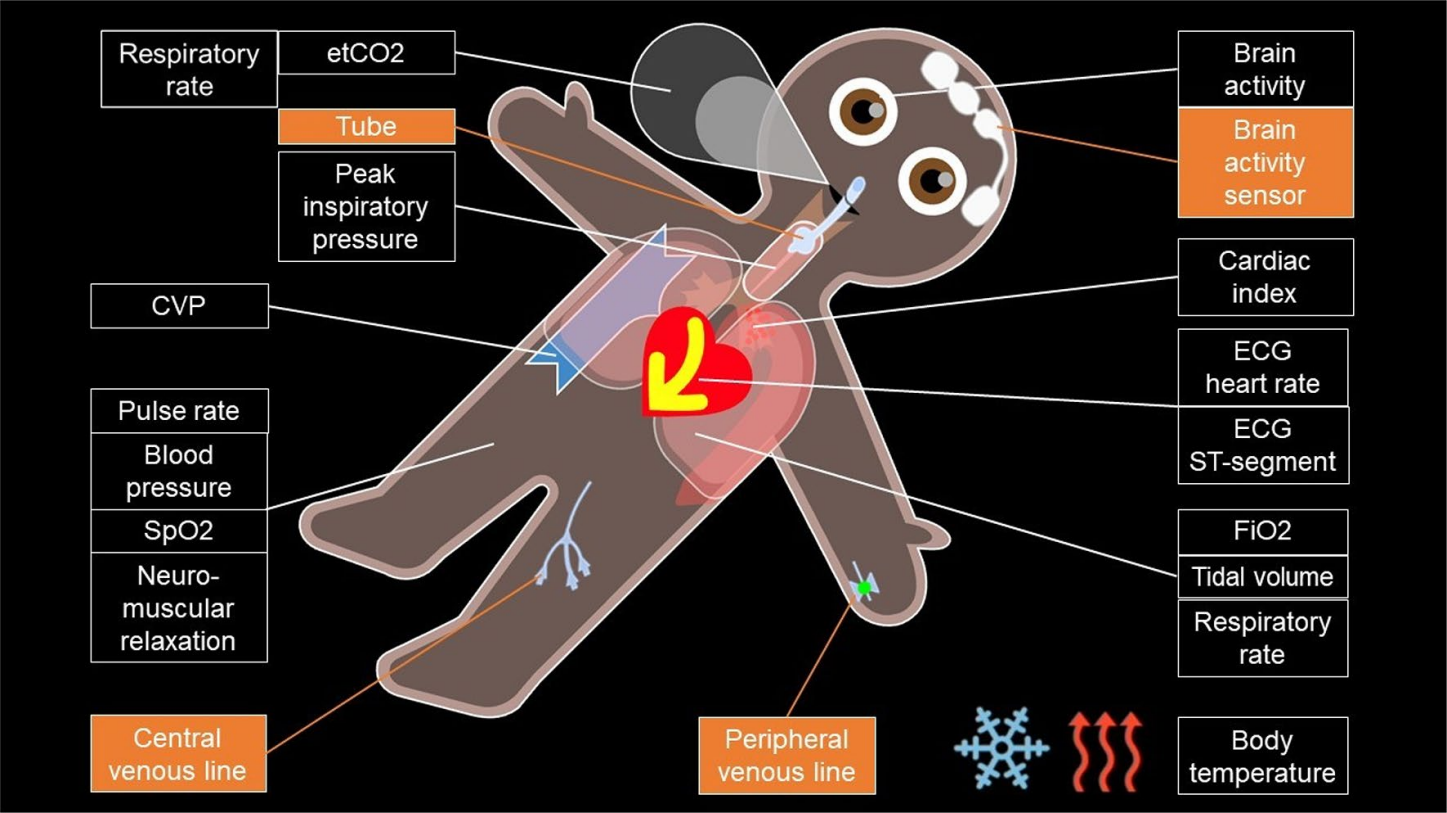
Visual-Patient-avatar ICU displaying the fifteen currently available vital signs and selected installations (orange boxes). When body temperature deviates from the normal range, heat waves or ice crystals appear around the avatar. *CVP* central venous pressure, *ECG* electrocardiogram, *etCO2* end-tidal carbon dioxide, *FiO2* inspiratory oxygen concentration, *SpO2* peripheral oxygen saturation. Additional File 1 shows all available installations as a table. Pulse rate and ECG heart rate were merged and counted as one item in the study.

Visual-Patient-avatar displays the status of a patient’s vital signs in real-time by modifying the shape, colour, animations and icons. For example, if body temperature exceeds a specific value, heat waves appear around the avatar, while during measured hypoxemia, the skin colour turns purple. The latest development step, the Visual-Patient-avatar ICU, enables the display of patient installations, presented on the avatar as realistic icons at their respective insertion points. Figure 1 shows the fifteen currently available vital signs and installations eclectic in Visual-Patient-avatar ICU.

### Study design and participants

This was an investigator-initiated, prospective, multicentre, computer-based, within-subject study comparing two different patient monitor modalities (Visual-Patient-avatar ICU versus conventional monitor) in various patient scenarios. The data collection took place in five study centres, all tertiary care hospitals: University Hospital of Zurich and Hirslanden Clinic of Zurich in Switzerland, University Hospital of Frankfurt and University Hospital of Wuerzburg in Germany and Hospital Clinic de Barcelona in Spain. We recruited five nurses and five physicians at each centre based on their availability from clinical work, yielding 50 participants. All of them regularly work in ICUs and/or keep intensive care board certifications in their respective country. Participation was voluntary and without financial compensation. The participants who attended the pilot study in May 2021 at the University Hospital of Zurich were excluded.

### Study procedure

**Figure 2:**
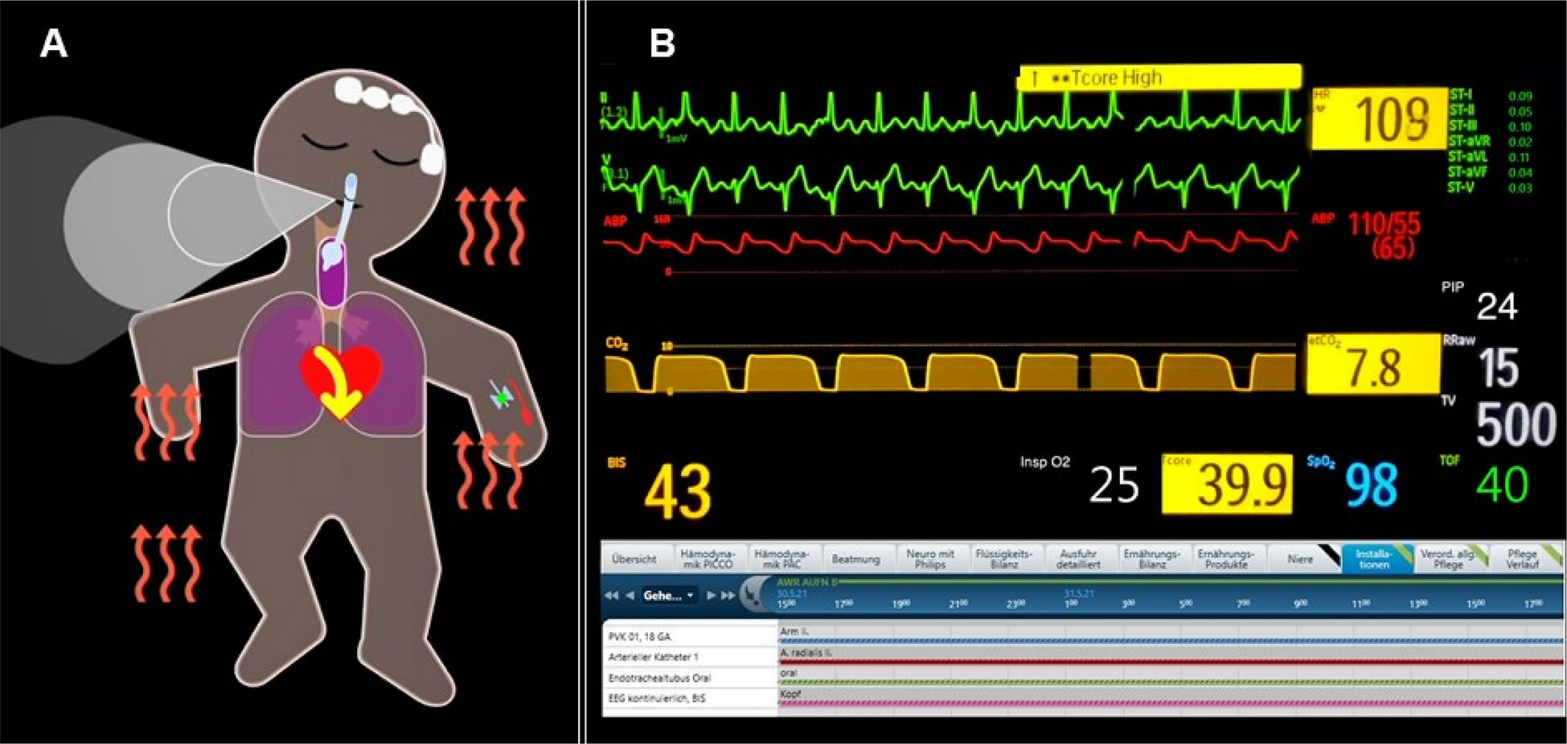
Exemplary scenario depicted in both modalities. (**A**) Visual-Patient-avatar ICU and (**B**) the corresponding conventional patient monitor used in Switzerland. For example, the large extension of the CO_2_ cloud designates too high end-tidal carbon dioxide, while the floppy extremities indicate a deep neuromuscular relaxation. Tachycardia is only visible in the animated image as rapid pulsations. We adjusted the units and labellings’ language of the conventional patient monitor country specifically. *etCO*_*2*_ end-tidal carbon dioxide, *HR* Heart rate, *Tcore* Core temperature, *PIP* Peak inspiratory pressure, *ABP* Arterial blood pressure, *TV* Tidal volume, *TOF* Train of four ratio, *Insp O*_*2*_ Inspiratory oxygen concentration, *BIS* Bispectral Index, *RRaw* Respiratory rate, *SpO*_*2*_ peripheral oxygen saturation.

We prepared five scenarios of possible patient conditions with varying vital sign deviations and installations (Additional File 1). Each scenario was shown twice, once as Visual-Patient-avatar ICU and once as matching conventional monitor modality resulting in ten cases. Figure 2 depicts exemplary scenario number three in both modalities. We created a unique case sequence for each participant using ResearchRandomizer version 4.0 (http://www.randomizer.org) and prepared corresponding PowerPoint presentations. We used a Dell Alienware laptop (Dell Technologies Inc., Round Rock, TX, USA) or an Apple MacBook (Apple Inc., Cupertino, CA, USA) for demonstration.

The study followed a standardised procedure, including an introduction, a teaching video on Visual-Patient-avatar ICU (Additional File 2) and an example of the conventional monitor modality for familiarisation. All participants received a table showing the defined reference values of the vital signs (Additional File 3). Participants` task was to remember the status of the vital signs, which could be too low, safe, too high or not measured, as well as the type and location of present installations. Before starting the data collection, open questions were answered and a brief demographic survey was completed. After showing each case for fifteen seconds, the participants entered their answers in a pre-programmed survey with checkboxes on an iPad (Harvest your data, Wellington, New Zealand, and Apple Inc., Cupertino, CA, USA). Thereby, "no recall" could be selected as a further option. After each of the ten cases, participants self-rated their diagnostic confidence and perceived workload in fulfilling the given task. At the end of data collection, all participants were invited to evaluate four general statements on a 5-point Likert Scale (from strongly disagree to strongly agree) to gain general subjective impressions about the Visual-Patient-avatar ICU monitor modality. Additional File 4 contains a PowerPoint presentation, including the introduction and one case as an example.

### Outcome parameters

The study’s primary outcome was information transfer measured by performance in assessing vital sign status and present installations. In total, 22 different items (Additional File 1) were tested. These answers were evaluated binary, whether correct or incorrect. The option “no recall” was counted as an incorrect answer. Secondary outcome parameters were participants’ evaluations of their diagnostic confidence on a 4-point Likert scale (from very unconfident to very confident) and perceived workload using the National Aeronautics and Space Administration Task Load Index (NASA-TLX) to take into account human factors as well^5,6,25^. Latter is a validated tool for workload self-assessment containing six sub-questions. We removed one question because of the absence of physical demand^26–28^. The participants rated the five questions numerically from 0 (very low) to 100 (very high).

### Statistical analysis

For descriptive statistics of continuous data, medians were presented with interquartile ranges (IQR) and means with standard deviations (SD). McNemar’s test was applied for an unadjusted comparison of the binary primary outcome variable between the two modalities Visual-Patient-avatar ICU and conventional monitor. Crude-matched odds ratios investigated the correct responses of each vital sign and installation tested in Visual-Patient-avatar ICU. To take into account dependencies due to repeated measurements, we calculated mixed Poisson regression for the number of correct answers with a random intercept for each participant, including potential covariates like age, gender, profession, scenario and study centre.

Regarding the secondary outcome of diagnostic confidence, a binary variable was created by summarising the options “very confident and confident” and “very unconfident and unconfident” into one value each. A mixed logistic regression model was used to quantify differences between the two modalities regarding feeling confident in solving a task.

Further, we assessed the NASA-TLX as a surrogate for perceived workload with a linear mixed model. For this enquiry, the five sub-questions results were merged into an overall score by calculating the arithmetic mean. Both mixed models contained a random intercept per participant and were adjusted for the same covariates as the Poisson model mentioned above. Applying the one-sample Wilcoxon signed-rank test, we evaluated if the distribution of the answers to the general statements was symmetrical around the median (the neutral answer) or whether there was a tendency towards approval or rejection.

Outset data processing was performed with Microsoft Excel (Microsoft Corporation, Redmond, Washington, USA). For data analysis, we used R version 4.0.5 (R Foundation for Statistical Computing, Vienna, Austria) and IBM SPSS Statistics 26 (International Business Machines Corporation, Armonk, New York, USA). Statistical significance was set at *P* < 0.05. To create figures and graphs, Microsoft PowerPoint (Microsoft Corporation, Redmond, Washington, USA) and GraphPad Version 8.4.3. (GraphPad Software Inc., California, USA) were used.

### Sample size calculation

In May 2021, a pilot study with seven participants at the University Hospital Zurich was conducted, revealing a rate ratio of correct decisions of 1.55 in Visual-Patient-avatar ICU compared to the conventional modality. A smaller effect with a rate ratio of 1.25 was presumed to determine the final sample size. Assuming a significance level of 5% and a power of 90%, we calculated a minimum sample size of eight participants. Due to the multicentre study design and the fact that no nurses took part in the pilot study, we decided to include five nurses and five physicians in each study centre.

### Ethics approval and consent to participate

This project did not fall within the Human Research Act’s scope. According to the local boards, ethical approval was not necessary for all study centres in Switzerland, Germany and Spain.

Written informed consent was obtained from the participants before the start of the study.

## Results

Between June 2021 and August 2021, we recruited 50 participants in the five study centres. The participants completed in total 500 cases, 250 with Visual-Patient-avatar ICU and 250 corresponding with the conventional monitor modality. Each case contained 22 items, resulting in 11,000 individual ratings. No ratings had to be excluded except for one answer to the general statements. Table 1 provides detailed information about participant characteristics.

**Table 1:** Participant characteristics (N = 50). Values are numbers (proportion) or median (IQR^29^). We counted the years after university graduation (physicians) or nursing education (nurses) as work experience. *IQR* interquartile range.

Characteristics	Physicians, n = 25	Nurses, n = 25	Combined, N = 50
Age in years	36.5 (32.8–43.3 [28–53])	37 (33.0–43.8 [27–56])	37.0 (33.0–43.8 [27–56])
Work experience in years	10 (7.0–16.3 [4–28])	10.5 (7.3–16.8 [5–37])	10.5 (7.2–16.8 [4–37])
Gender female, n (%)	7 (28%)	12 (48%)	19 (38)

### Primary outcome: performance in assessing vital sign status and installations

**Figure 3:**
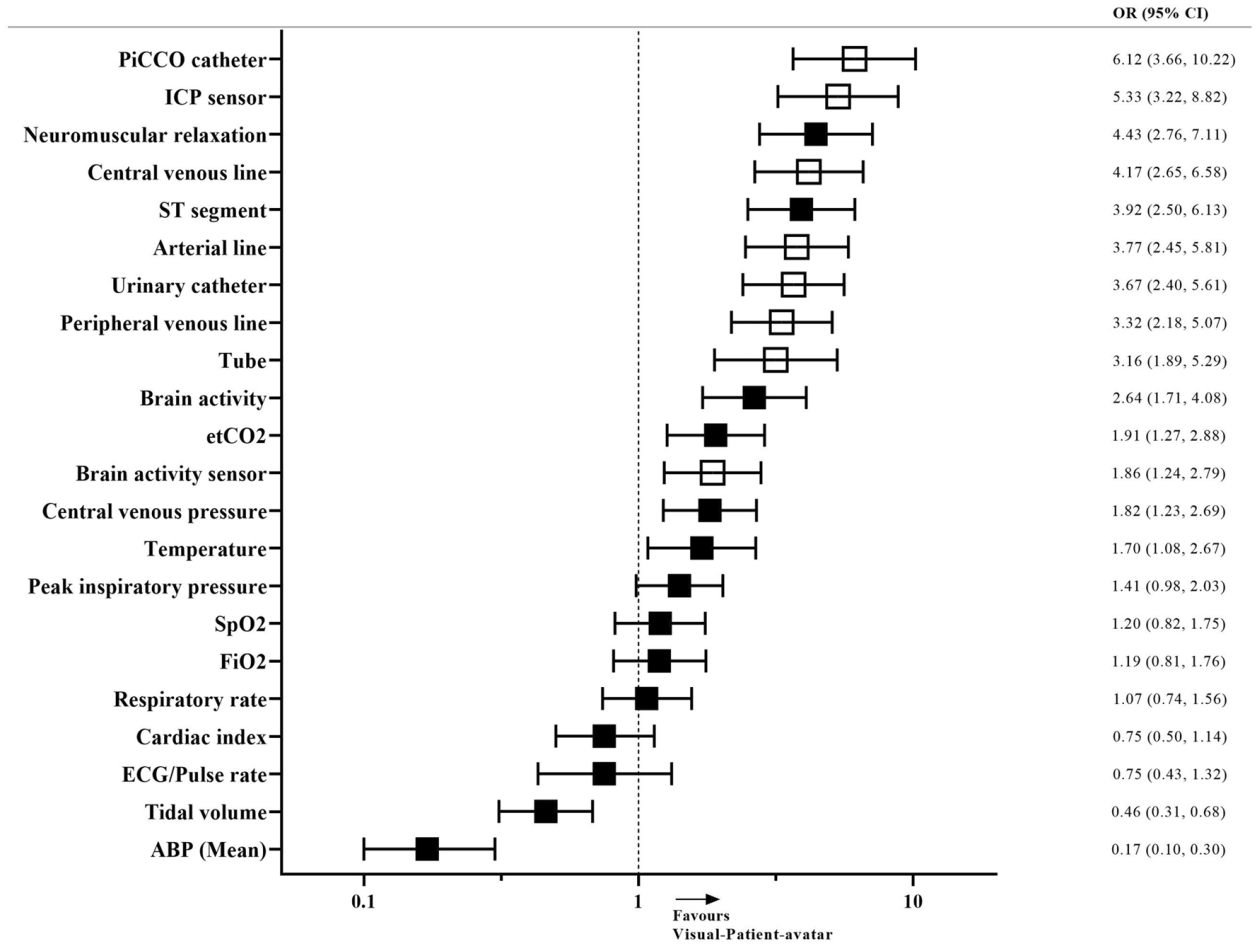
Matched odds ratios of the different vital signs and installations for Visual-Patient-avatar ICU and conventional monitor. Odds ratios (OR) are presented by boxes and whiskers indicate the 95% confidence interval. Filled boxes represent vital signs, while empty boxes illustrate installations. *ICP* Intracranial pressure, *etCO*_*2*_ end-tidal carbon dioxide, *SpO*_*2*_ peripheral oxygen saturation, *FiO*_*2*_ inspiratory oxygen concentration, *ABP* Arterial blood pressure.

Concerning the primary outcome, binary performance in assessing vital sign status and installations, the descriptive analysis revealed a higher number of correct decisions using Visual-Patient-avatar ICU (14.1 of 22 [3.3], mean [SD]) than conventional modality (11.3 of 22 [3.4], mean [SD]). The unadjusted comparison of binary primary outcome data by McNemar’s test disclosed very strong evidence (*P* < 0.001) favouring the visualisation technique. Concluding, the mixed Poisson regression model confirmed the difference between the two modalities with very strong evidence. The rate of correct decisions was about 1.25 times higher using Visual-Patient-avatar ICU than the conventional monitor [rate ratio (RR) 1.25; 95% CI 1.19–1.31; *P* < 0.001]. For a more profound investigation of the primary outcome, we analysed the vital sign- and installation-related questions separately with further mixed Poisson regression models. The first mixed model assessed more correct vital signs with Visual-Patient-avatar ICU (RR 1.1; 95% CI 1.03–1.17; *P* = 0.003). The calculation in terms of the installation-related questions showed even very strong evidence for an effect of the modality. With Visual-Patient-avatar ICU, the rate of correctly assessed installations was about 3.32 times higher compared to the conventional monitor (RR 3.32; 95% CI 2.15–5.11; *P* < 0.001). We tabulated the influence of the covariates age, gender, profession, scenario and study centre in all three mixed Poisson regression models (Additional File 5). Subsequently, calculated crude matched odds ratios demonstrated that the participants rated 18 of the 22 primary outcome items better with Visual-Patient-avatar ICU. Figure 3 presents this descriptive analysis of all vital signs and installations tested in detail.

### Secondary outcomes: diagnostic confidence and perceived workload

The analysis of diagnostic confidence by a mixed logistic regression model showed very strong evidence that the participants were more confident when using Visual-Patient-avatar ICU (OR 3.32; 95% CI 2.15–5.11, *P* < 0.001). No strong statistical evidence for an effect of the covariates tested (Additional File 5) was found.

**Figure 4:**
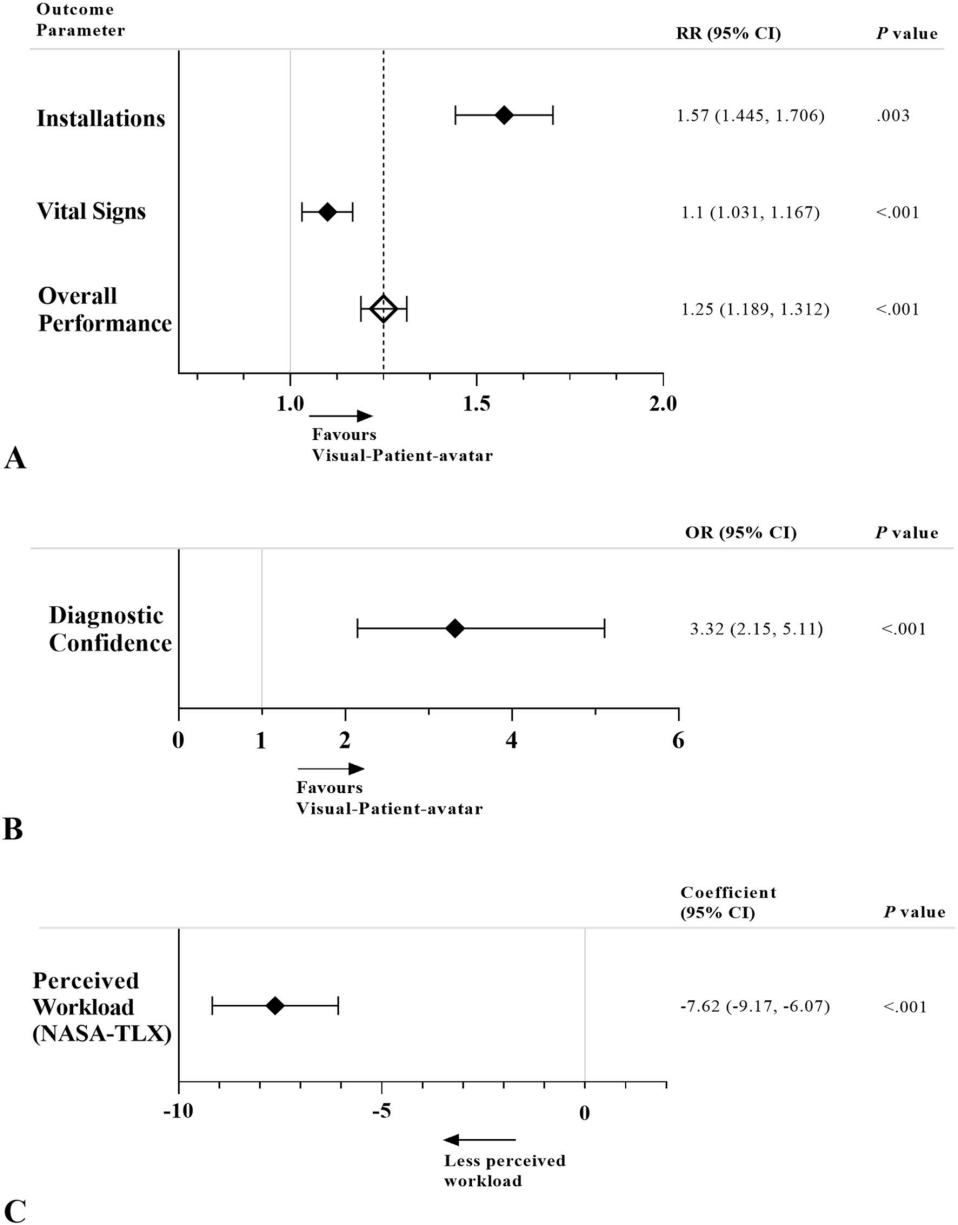
Graphical presentation of results for all outcome parameters. (**A**) Mixed Poisson regression model of correct answers relating vital signs and installations for Visual-Patient-avatar ICU and conventional monitor. Rate ratios (RR) are shown by squares and whiskers indicate the 95% confidence interval. (**B**) Mixed logistic regression model for diagnostic confidence of Visual-Patient-avatar ICU and conventional monitor. Odds ratio (OR) is shown by square and whiskers indicate the 95% confidence interval. (**C**) Mixed linear regression model for perceived workload of Visual-Patient-avatar ICU and conventional monitor. The coefficient is shown by square and whiskers indicate the 95% confidence interval. *NASA-TLX* National Aeronautics and Space Administration Task Load Index.

Finally, the linear mixed model examining perceived workload yielded very strong evidence for a difference between the two patient monitoring modalities. The overall NASA-TLX ratings were on average 7.62 points lower with Visual-Patient-avatar ICU compared to the conventional modality (Coefficient − 7.62; 95% CI − 9.17 to − 6.07; *P* < 0.001). There was no evidence of a perceived workload difference depending on age, gender, profession, scenario and study centre (Additional File 5). Figure 4 graphically presents the results for all outcome parameters.

**Figure 5:**
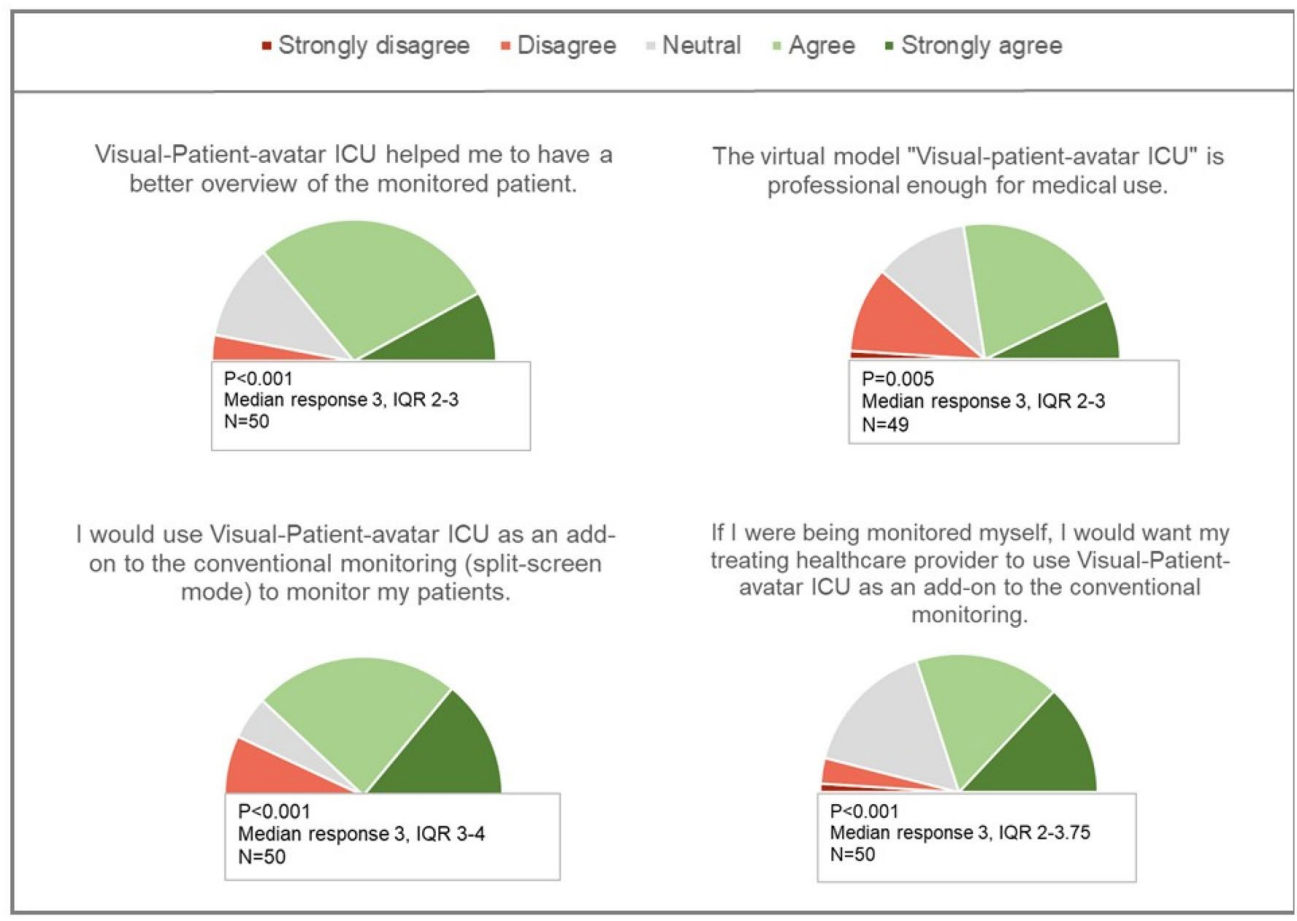
Graphical display of the participants’ assessment concerning the four general statements as half pie charts. Results are shown as median and interquartile range (IQR). We used Wilcoxon signed-rank test to determine whether the answers differ from neutral (P < 0.05).

Evaluation of participants’ rating concerning the four general statements applying the one-sample Wilcoxon signed-rank test revealed statistically significant approval of Visual-Patient-avatar (all P-values < 0.05), which is illustrated in Fig. 5.

## Discussion

This computer-based study reports 250 within-subject comparisons of assessing five patient scenarios using two different monitor modalities-Visual-Patient-avatar ICU and conventional monitor.

### Primary outcome: performance in assessing vital sign status and installations

Our results indicate an improved information transfer as the visualisation technique allowed for a higher rate of correctly retrieved vital signs and installations. Overall, Visual-Patient-avatar ICU enabled participants to remember 18 of the 22 primary outcome items better than the conventional modality. This was accompanied by strong evidence for Visual-Patient-avatar ICU increasing diagnostic confidence and lowering perceived workload. Finally, the participants esteemed the overview and professional design provided by the avatar. They agreed that Visual-Patient-avatar ICU is a valuable add-on for patient monitoring in clinical practice.

With the extended Visual-Patient-avatar ICU, information transfer performance was significantly improved compared to the conventional monitor, consistent with previous studies of the Visual-Patient-avatar for anaesthesia purposes^30–32^. The graphic avatar facilitates perception and comprehension by transforming variables into intuitive objects, designed to have a logical commonality with the reality they depict, thereby matching users’ mental models of vital signs or installations^21,24^. This approach follows user-centred design principles for fostering situation awareness, as the information transfer is quick with low cognitive demand^21,33,34^. These results support the idea that considering human factors in designing patient data displays may help decrease human error by freeing up mental capacity and coping with information overload^13,35^. Other techniques merging alphanumeric patient data into a visualisation, like the Dynamic Lung (Hamilton Medical AG, Bonaduz, Switzerland) already found their way into clinical practice^17,18,36^.

The more profound analysis of the primary outcome data revealed that with Visual-Patient-avatar ICU, the rate of correctly recognised vital signs as well as installations was higher, with a more pronounced effect for installations. Only two of the 22 items tested, arterial blood pressure and tidal volume, were retrieved significantly better with the conventional modality, providing valuable insights for possible design improvements.

### Secondary outcomes: diagnostic confidence and perceived workload

Besides the clear benefits of Visual-Patient-avatar ICU for information transfer, the participants indicated higher diagnostic confidence and less perceived workload. Both low confidence and increased workload are psychological stressors^21,25,37^. Stress can cause cognitive changes, which may impair attention or deteriorate information recall leading to reduced task performance^9,21,37,38^. These are significant findings, mainly because the participants were Visual-Patient-avatar ICU novices. They indicate that the implementation of user-centred design principles succeeded as such systems are created around users’ needs^34^. Contrary, it is possible that the avatar improved diagnostic confidence and perceived workload since less complex information is presented through its categorised (too low, safe or too high) and thus simplified design of data display. Here is to counter that the benefits may be even underestimated as the participants just got a short training video on Visual-Patient-avatar ICU for familiarisation compared to their vast experience with conventional modality. These results support the idea of avatar-based patient monitoring as valuable but not burdening additional information elements complementing the conventional modality by guiding the attention to the problem at hand. A high-fidelity simulation study among anaesthesia personnel examining a split-screen display presenting both modalities side by side revealed non-inferiority of this innovative patient monitoring and earlier communication of the clinical diagnosis^22^.

In this study, the subjective survey completing the objectively verifiable data revealed great approval of the visualisation technique. Regarding the analysis of the general statements about Visual-Patient-avatar ICU, a statistically significant part of participants agreed or strongly agreed that its design is professional enough for medical use. Furthermore, they confirmed Visual-Patient-avatar ICU’s usability as an add-on to conventional modality in a real-life setting and appreciated the overview provided. Used in split-screen mode, the avatar’s strength can particularly take effect as it improves situation awareness for possible changes quantifiable with the conventional modality.

### Strengths and limitations

The study has the inherent limitations of computer-based studies. We prepared theoretical patient conditions and presented them in an artificial environment that did not correspond to the actual location of patient monitoring. In addition, the data collection occurred in disregard of clinical context like the patient’s history. Thereby results may differ in a real-life setting. Nevertheless, this concept allowed the new Visual-Patient-avatar ICUs’ features to be tested in a safe and standardised environment, which existing literature recommends and attenuates unknown confounders^39,40^. These facts essentially influenced our further study planning since we intend the visualisation technique’s clinical implementation in selected centres. The information on perceived workload is subjective in nature. Despite the use of the validated NASA-TLX score, it is possible that this information may vary and could be different, especially in the clinical setting. This study also has decided strengths. The study’s international multicentre design mitigates local biases and allows the generalisation of our findings. Regarding participants’ characteristics, the selection provides a vast range of ages and work experience, including two occupational groups. The study’s internal validity is high as each participant handled all five patient scenarios in both monitoring modalities in a randomised sequence. Finally, the sample size selected was based on calculations drawn from a pilot study and guaranteed adequate analysis power.

## Conclusion

In this computer-based study, Visual-Patient-avatar ICU improved information transfer and diagnostic confidence while reducing perceived workload of nurses and physicians working on different patient scenarios compared to the conventional monitor modality. These findings emphasise the crucial impact of user-centred design features on performance and subjective perception. The avatar’s principles seem to foster the process of perception and comprehension of vital signs and installation data, possibly leading to better decision-making with less cognitive effort among ICU personnel. The results highlight the potential of integrating diverse medical data into a single user interface to enhance patient safety and care. Medical personnel are as good as the information they receive.

## Supplementary Information

Supplementary Information 1.

Supplementary Video 1.

Supplementary Information 2.

Supplementary Information 3.

Supplementary Information 4.

## Data Availability

The datasets generated during the study are available from the corresponding author upon request.

## Abbreviations

CI: Confidence interval
ICU: Intensive care unit
IQR: Interquartile ranges
NASA-TLX: National Aeronautics and Space Administration Task Load Index
RR: Rate ratio
SD: Standard deviation
