# Supplementary material for: Avatar-based patient monitoring improves information transfer, diagnostic confidence and reduces perceived workload in intensive care units: computer-based, multicentre comparison study
Source: Sci Rep. 2023 Apr 11;13:5908. doi: 10.1038/s41598-023-33027-z (PMC10088750; doi:10.1038/s41598-023-33027-z)
Supplement: Supplementary file 1 — Supplementary Information 1. [file 41598_2023_33027_MOESM1_ESM.pdf]

Additional file 1. Qualitative overview of all scenarios with collaborating vital sign deviations and present installations. Orange= too low; green= safe; purple= too high; red= abnormal; grey= not measured/ none; blue= yes. Neuromuscular relaxation was measured by train of four (TOF)-ratio. Brain activity was measured by bispectral-index (BIS).

**Scenario**	**1**	**2**	**3**	**4**	**5**
**Vital sign**					
ECG/Pulse rate	Green	Green	Purple	Purple	Green
ABP (mean)	Purple	Orange	Green	Green	Green
CI	Grey	Orange	Grey	Purple	Grey
CVP	Green	Purple	Grey	Green	Grey
SpO2	Green	Orange	Green	Green	Orange
RR	Green	Green	Green	Purple	Green
TV	Purple	Green	Green	Grey	Orange
etCO2	Orange	Green	Purple	Grey	Purple
PIP	Green	Green	Green	Grey	Purple
FiO2	Green	Green	Orange	Grey	Purple
Temperature	Green	Green	Purple	Orange	Green
ST segment	Green	Red	Green	Red	Green
Neuromuscular relaxation	Relaxed	Not relaxed	Relaxed	Not relaxed	Not relaxed
Brain activitiy	Asleep	Awake	Asleep	Awake	Asleep
**Installation**					
Central venous line	Blue	Blue	Grey	Blue	Grey
Peripheral venous line	Blue	Grey	Blue	Blue	Blue
Arterial line	Blue	Grey	Blue	Grey	Blue
PiCCO® catheter	Grey	Blue	Grey	Blue	Grey
Tube	Blue	Blue	Blue	Grey	Blue
Urinary catheter	Blue	Blue	Grey	Blue	Grey
Intracranial pressor sensor	Blue	Grey	Grey	Grey	Grey
Brain activity sensor	Blue	Blue	Blue	Blue	Blue

Abbrevations: ABP: arterial blood pressure; ECG: electrocardiogram; CI: cardiac index; CVP: central venous pressure; SpO2: peripheral oxygen saturation; RR: respiratory rate; TV: tidal volume; etCO2: end-tidal carbon dioxide; PIP: peak inspiratory pressure; FiO2: inspiratory oxygen concentration; Tcore: core temperature; PiCCO: Pulse Contour Cardiac Output.
