# Supplementary material for: Avatar-based patient monitoring improves information transfer, diagnostic confidence and reduces perceived workload in intensive care units: computer-based, multicentre comparison study
Source: Sci Rep. 2023 Apr 11;13:5908. doi: 10.1038/s41598-023-33027-z (PMC10088750; doi:10.1038/s41598-023-33027-z)
Supplement: Supplementary file 3 — Supplementary Information 2. [file 41598_2023_33027_MOESM3_ESM.pdf]

Defined limits of vital signs

Parameter	Low VP	High VP	(Units)
**HR (ECG)**	<55	>100	/min
**Pulse**	<55	>100	/min
**ABP (Mean)**	<65	>100	mmHg
**NBP (Mean)**	<65	>100	mmHg
**CVP (Mean)**	<4	>12	mmHg
**STE**	<-0.2	>0.2	mV
**SpO2**	<94	none	%
**RR**	<8	>16	/min
**TV**	<350	>750	ml
**etCO2**	<35	>45	mmHg
**Tcore**	<36.0	≥37.5	°C
**BIS**	≤60	>60	(-)
**TOF rat.**	<90	≥90	%
**CI**	<2.5	>4	l/m^2/min
**Peak airway pressure (PIP)**	<10	>30	mbar
**Insp. O2**	<40	≥80	%
