# Supplementary material for: Avatar-based patient monitoring improves information transfer, diagnostic confidence and reduces perceived workload in intensive care units: computer-based, multicentre comparison study
Source: Sci Rep. 2023 Apr 11;13:5908. doi: 10.1038/s41598-023-33027-z (PMC10088750; doi:10.1038/s41598-023-33027-z)
Supplement: Supplementary file 4 — Supplementary Information 3. [file 41598_2023_33027_MOESM4_ESM.pptx]

## Slide 1
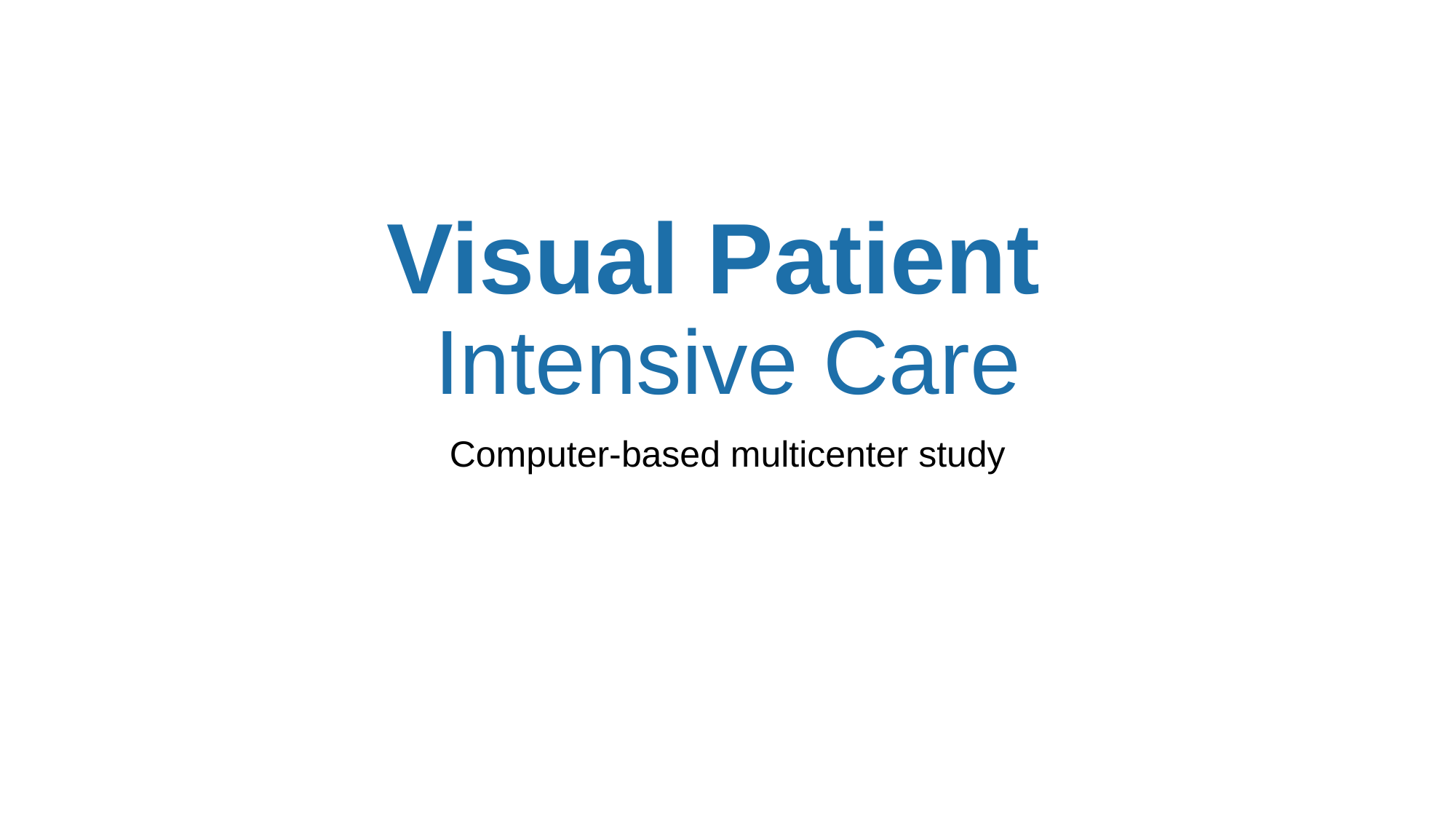

# Visual Patient Intensive Care
Computer-based multicenter study

## Slide 2
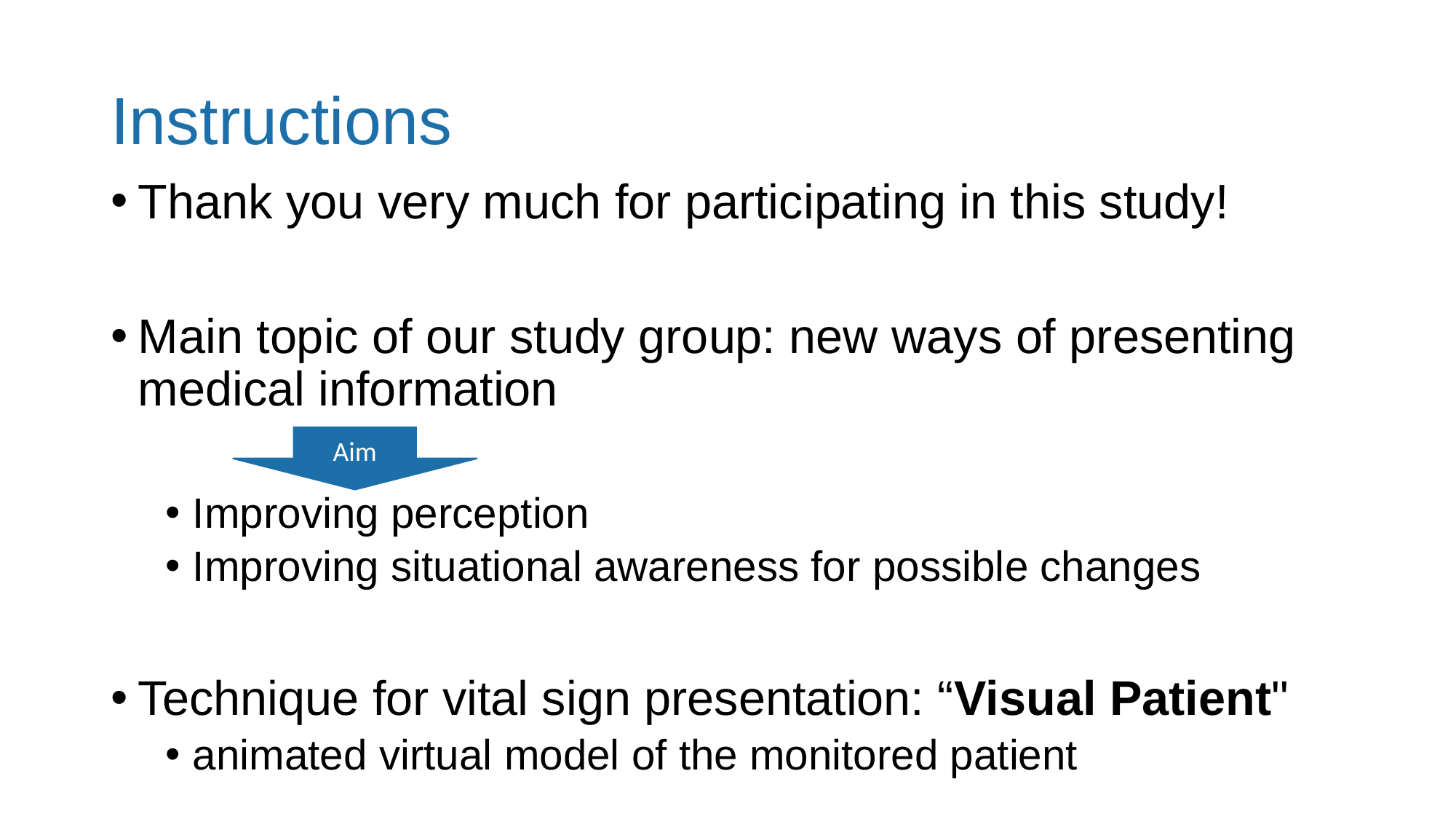

# Instructions
Thank you very much for participating in this study!
Main topic of our study group: new ways of presenting medical information
Improving perception
Improving situational awareness for possible changes
Technique for vital sign presentation: “Visual Patient"
animated virtual model of the monitored patient
Aim

## Slide 3
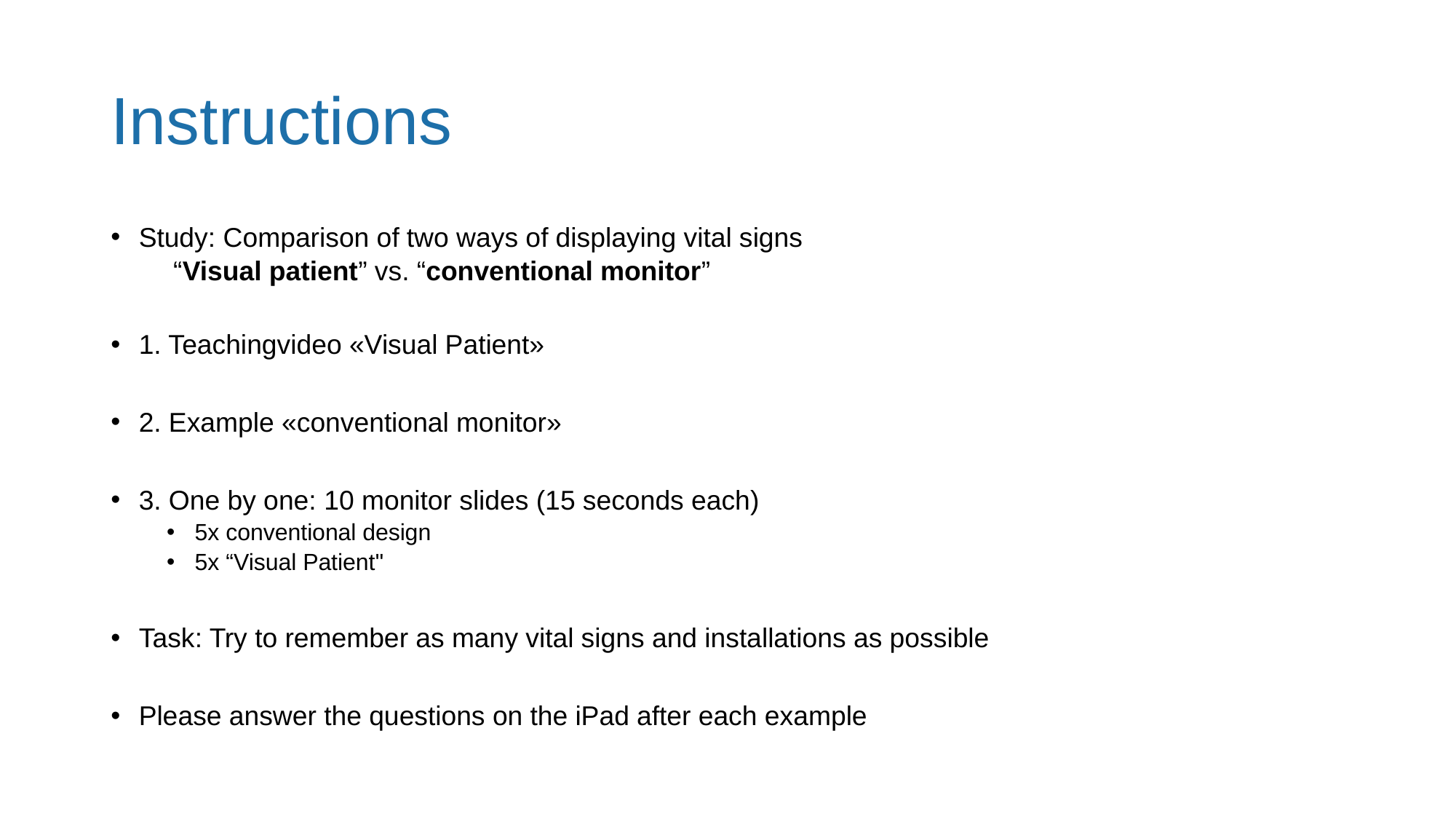

# Instructions
Study: Comparison of two ways of displaying vital signs
	 “Visual patient” vs. “conventional monitor”
1. Teachingvideo «Visual Patient»
2. Example «conventional monitor»
3. One by one: 10 monitor slides (15 seconds each)
5x conventional design
5x “Visual Patient"
Task: Try to remember as many vital signs and installations as possible
Please answer the questions on the iPad after each example

## Slide 4
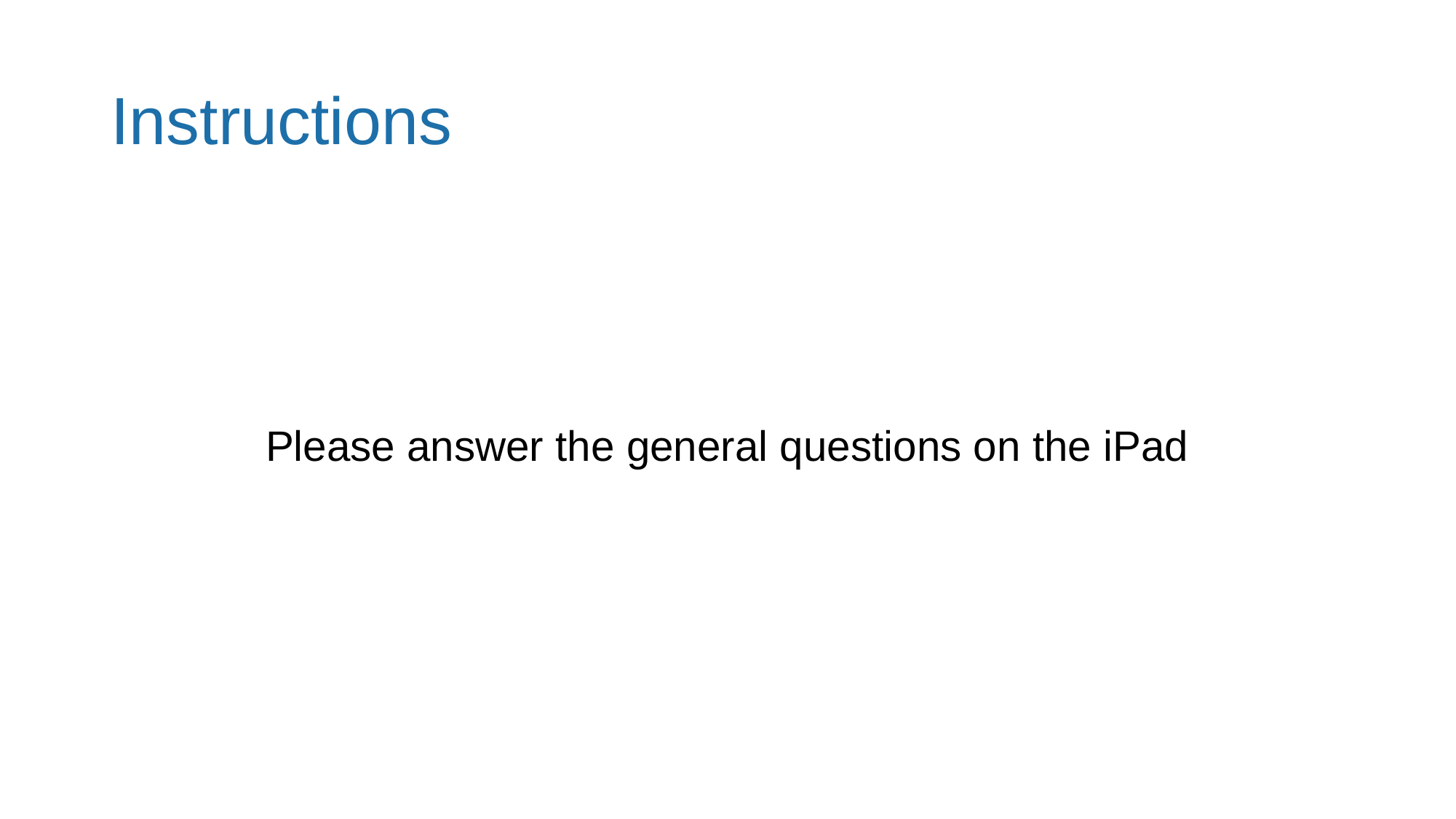

# Instructions
Please answer the general questions on the iPad

## Slide 5
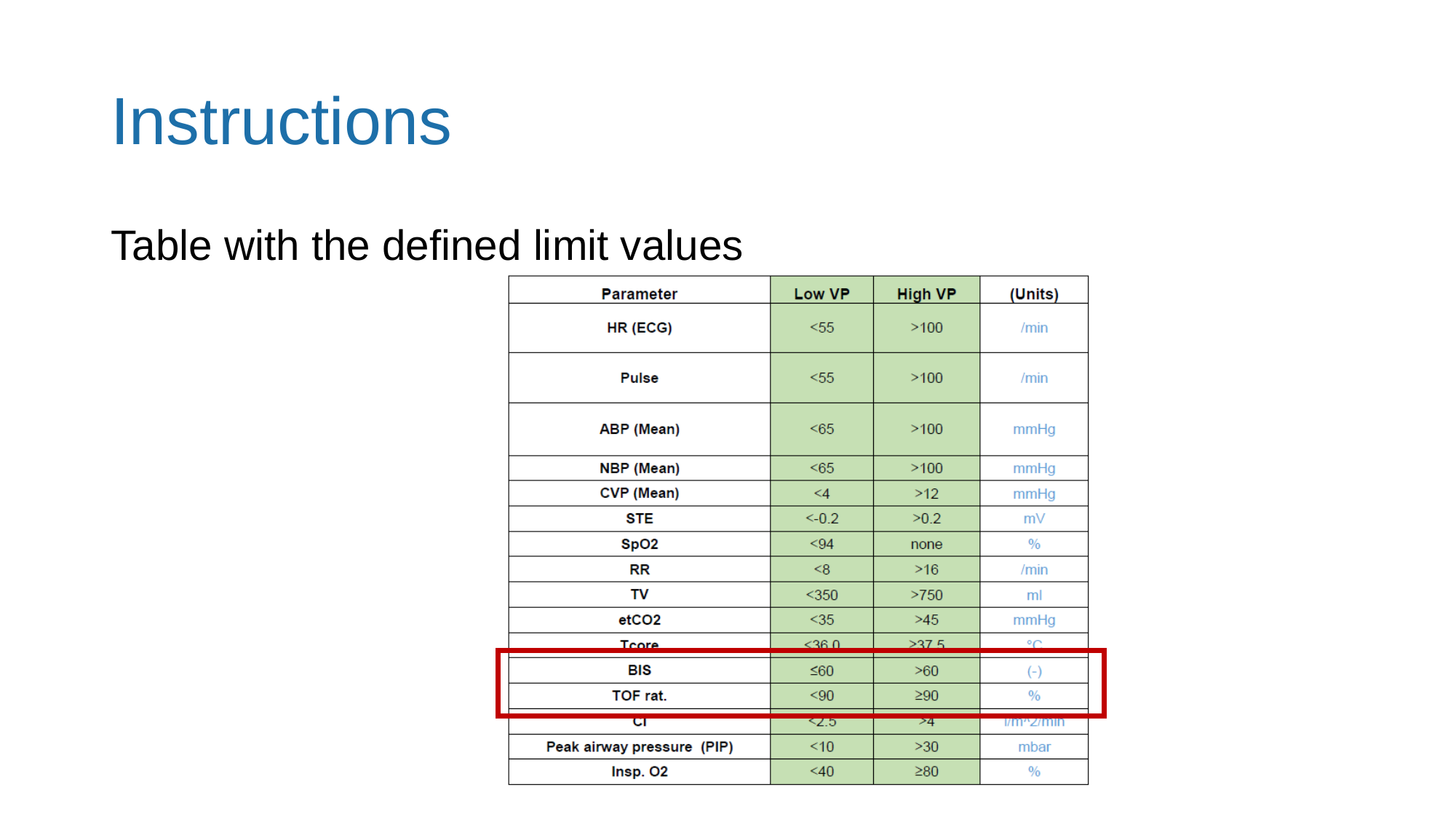

# Instructions
Table with the defined limit values

## Slide 6
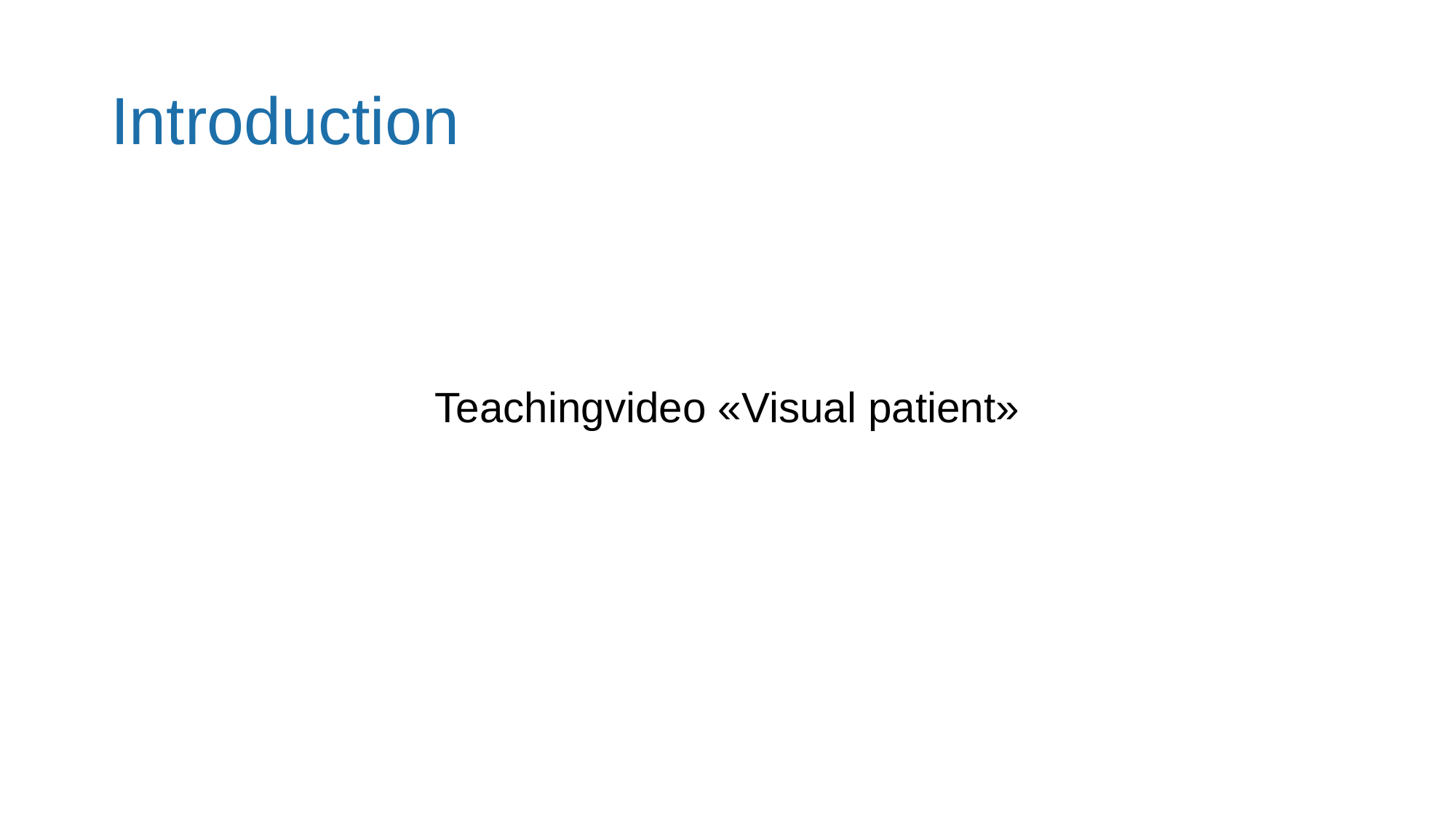

# Introduction
Teachingvideo «Visual patient»

## Slide 7
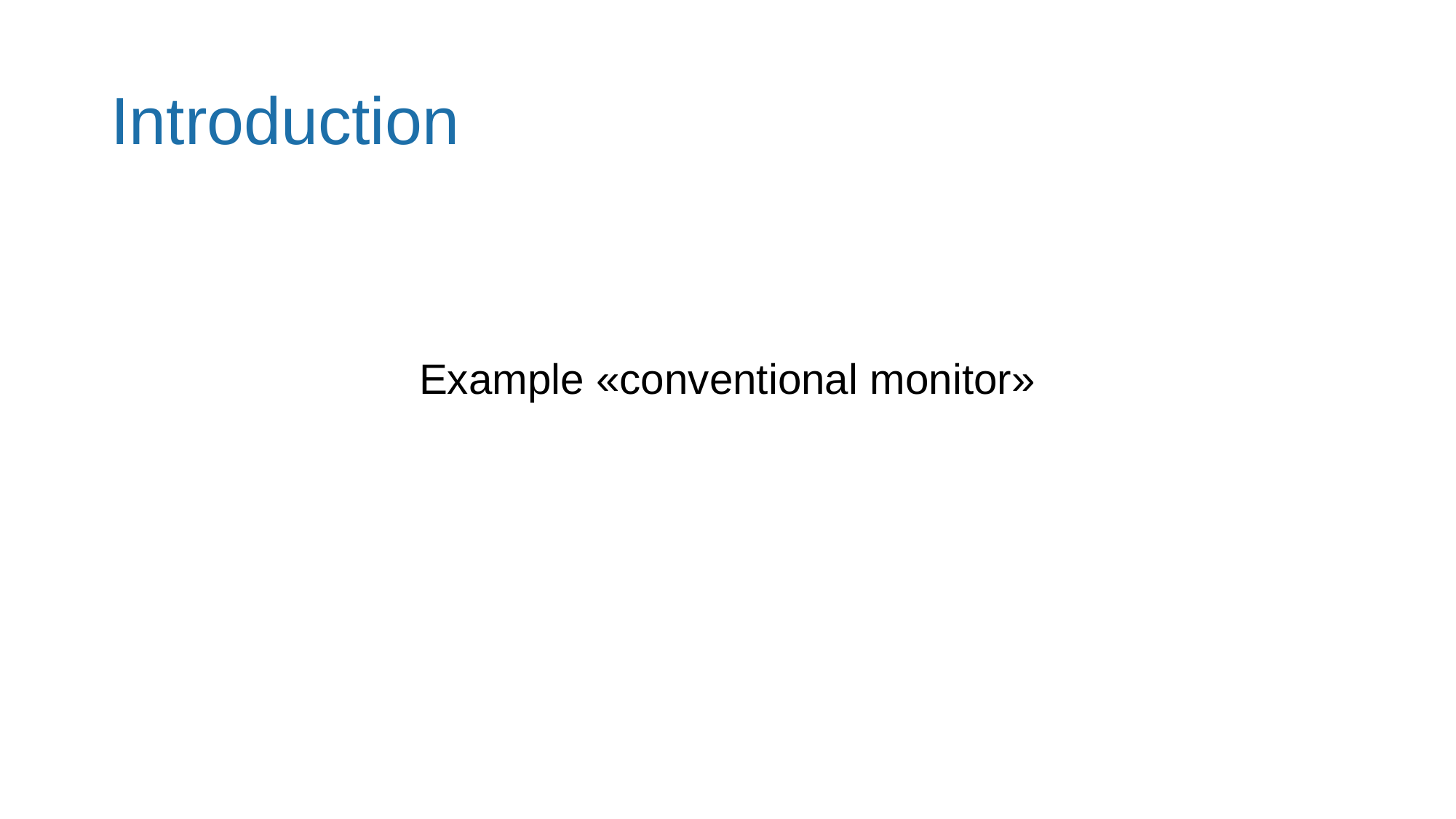

# Introduction
Example «conventional monitor»

## Slide 8
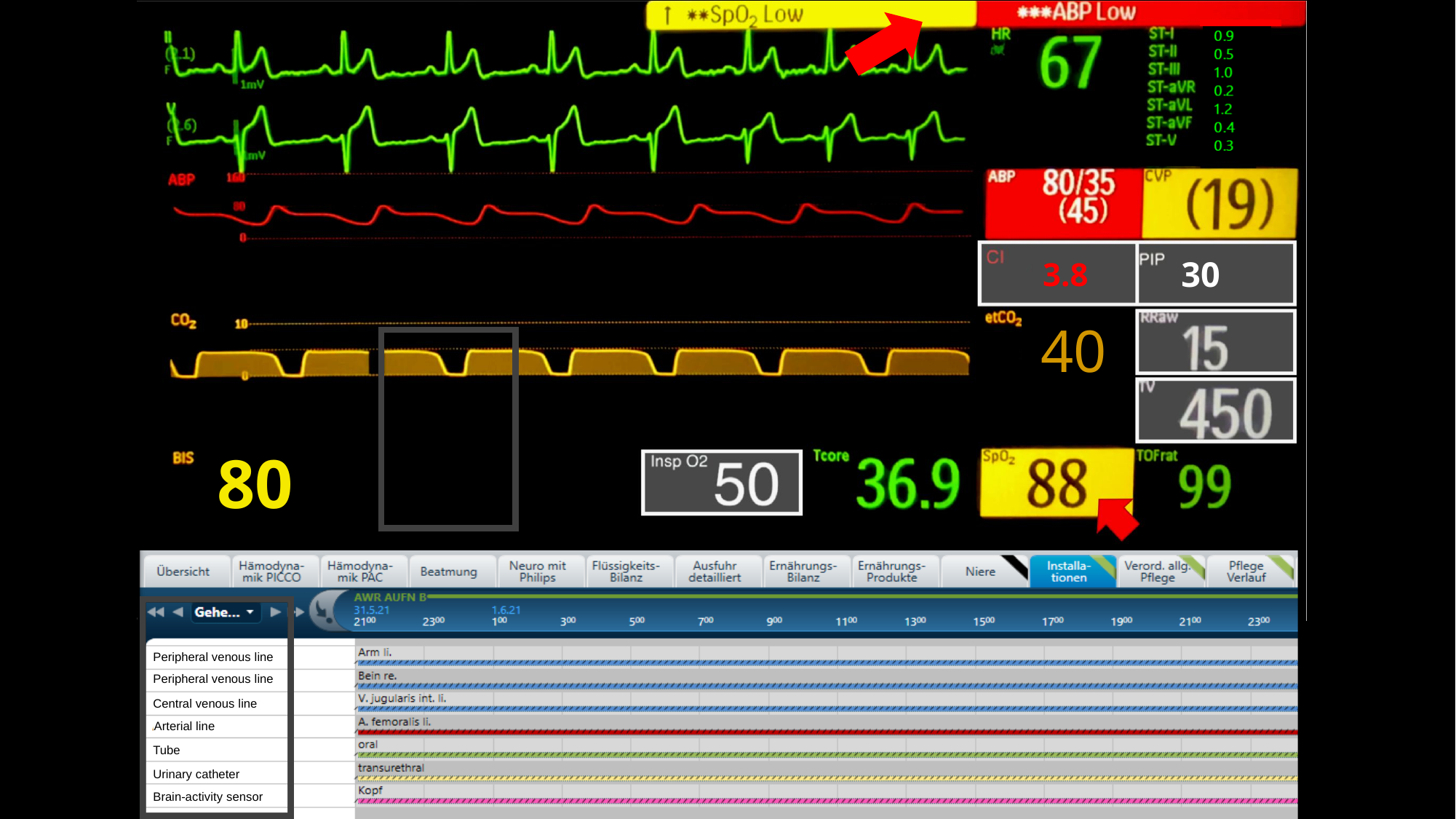

30
3.8
 40
60
80
Peripheral venous line
Peripheral venous line
Central venous line
Arterial line
Tube
Urinary catheter
Brain-activity sensor

## Slide 9
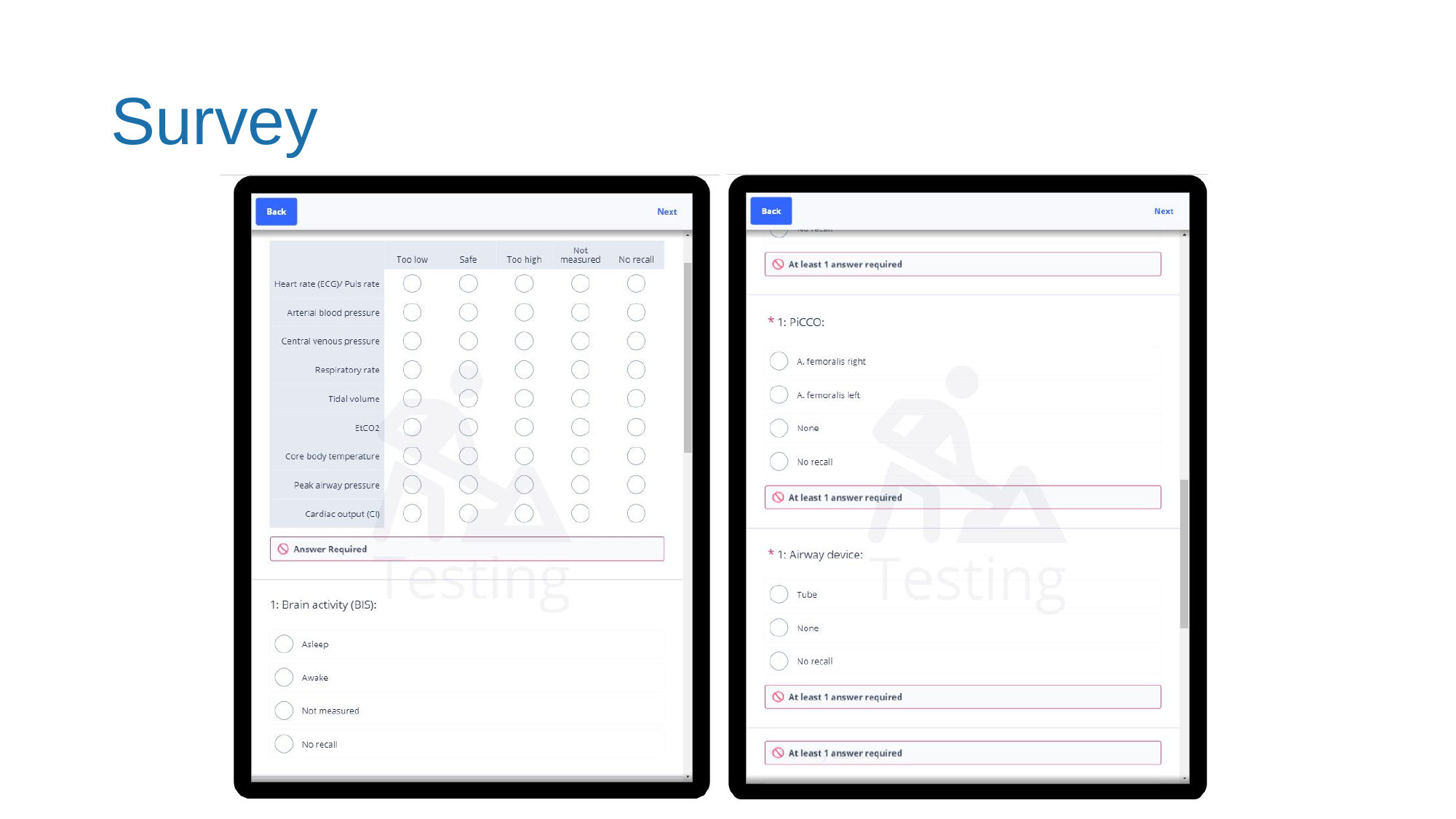

# Survey

## Slide 10
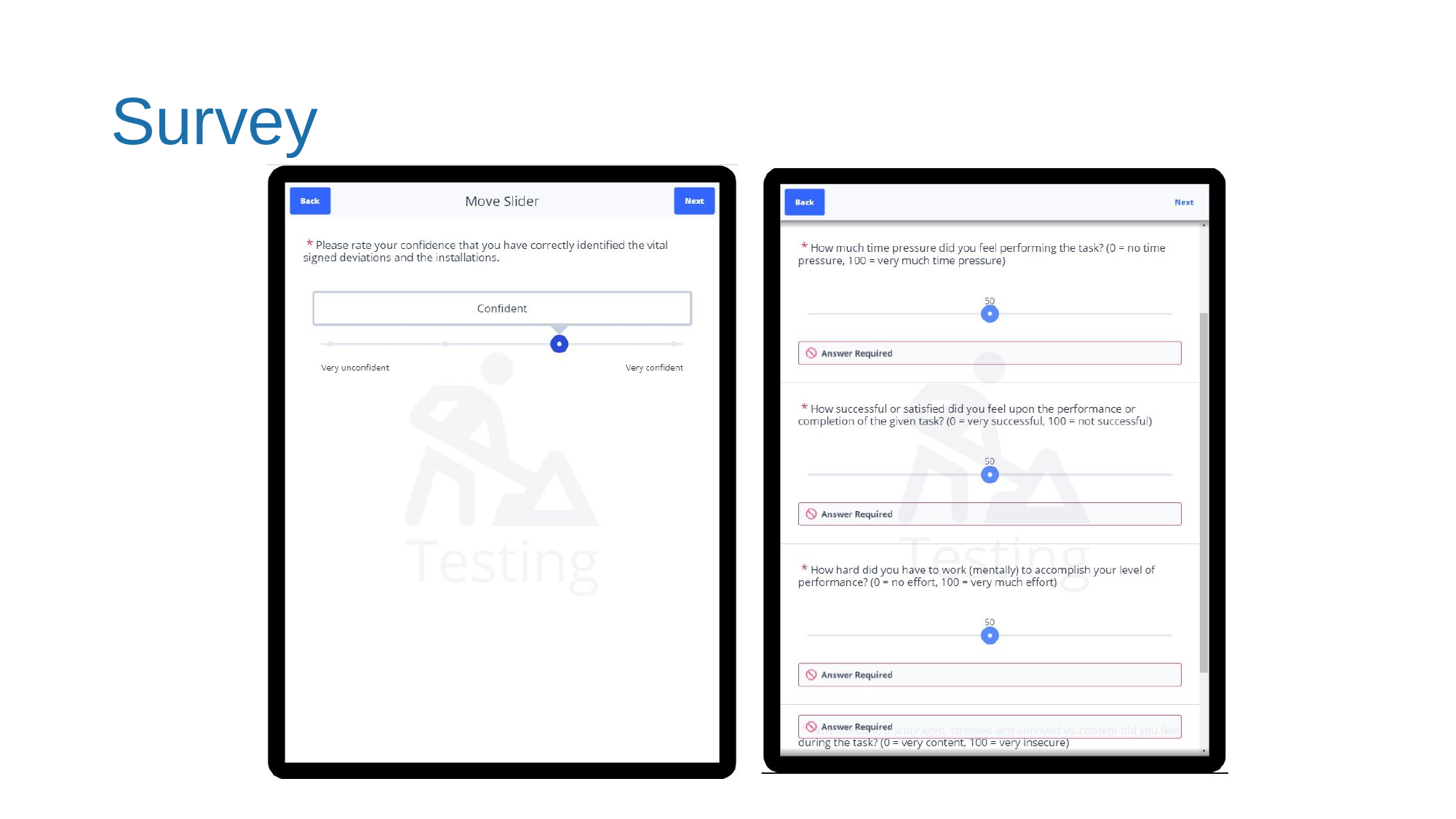

# Survey

## Slide 11
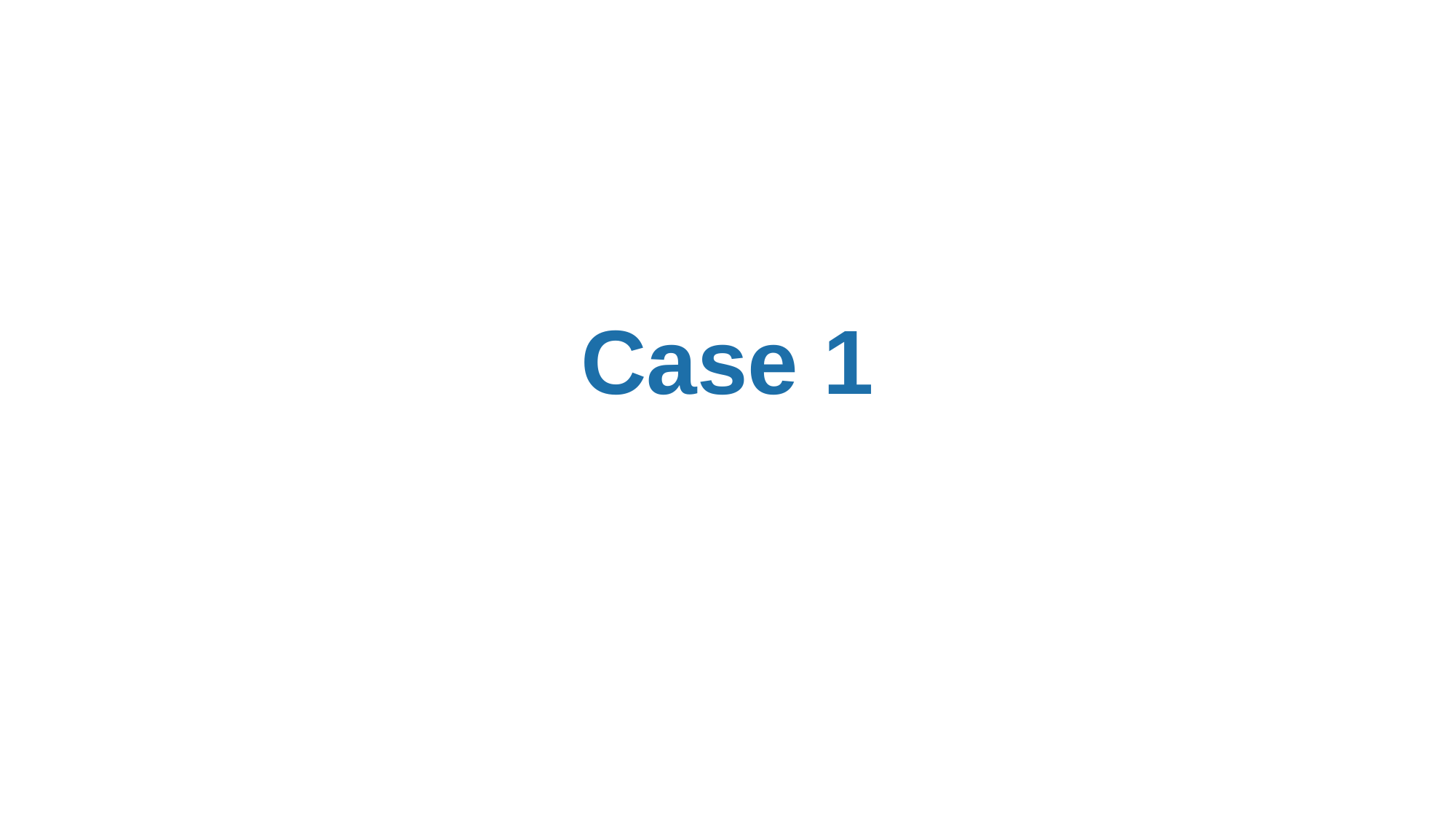

# Case 1

## Slide 12
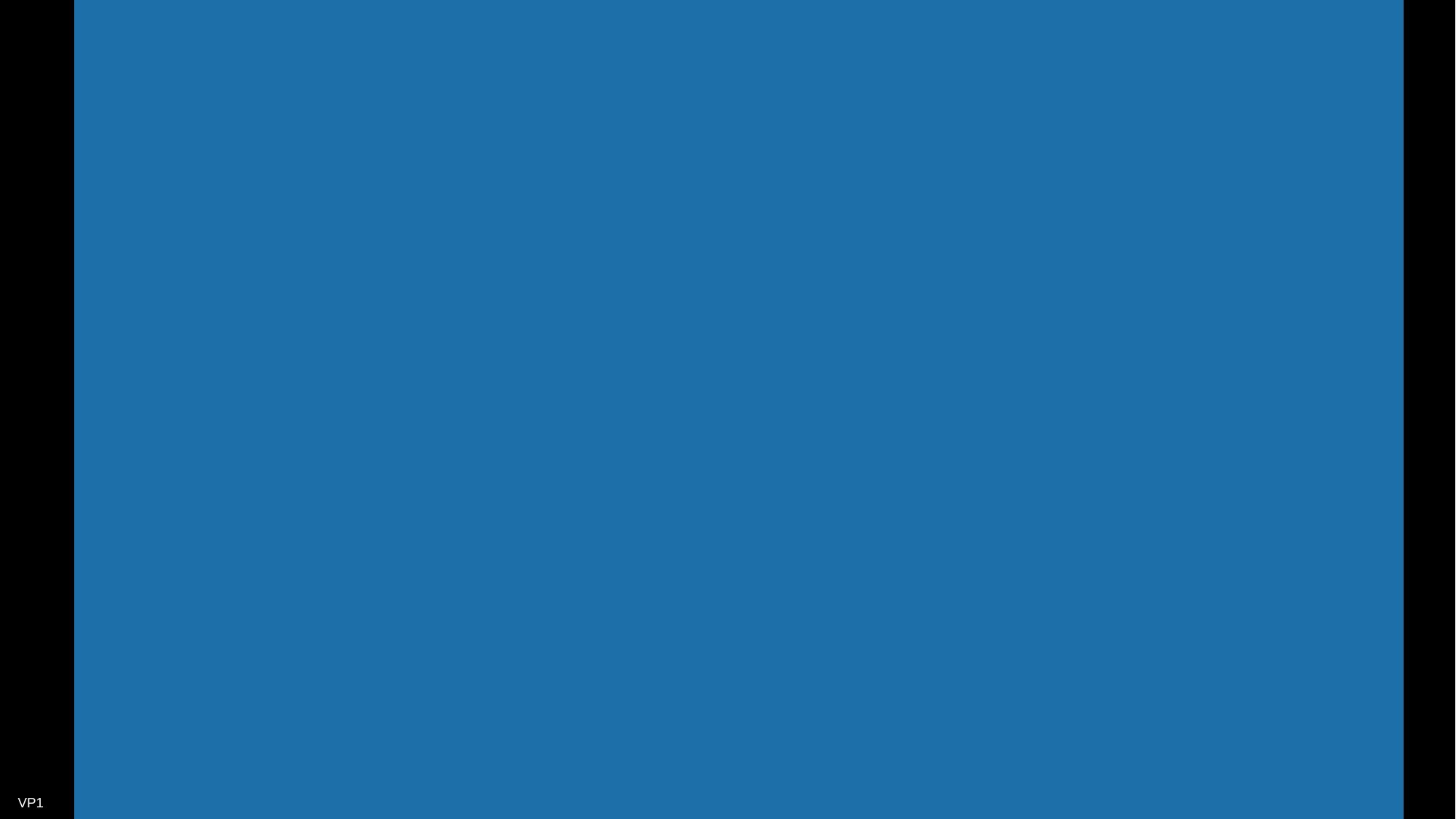

VP1
