# Supplementary material for: Avatar-based patient monitoring improves information transfer, diagnostic confidence and reduces perceived workload in intensive care units: computer-based, multicentre comparison study
Source: Sci Rep. 2023 Apr 11;13:5908. doi: 10.1038/s41598-023-33027-z (PMC10088750; doi:10.1038/s41598-023-33027-z)
Supplement: Supplementary file 5 — Supplementary Information 4. [file 41598_2023_33027_MOESM5_ESM.pdf]

Visual Patient ICU

Version 2.0
Julia Braun

November 4, 2021

Changes in comparison to the former version of this document:

- Job description as additional variable in the models
- Correction of mistake in regression tables (RR instead of OR)

1 Introduction

In this report, we compare the Visual Patient technology with the conventional monitor in an intensive care unit setting with respect to several different outcomes.

2 Methods

For descriptive statistics, we show means, standard deviations as well as medians and interquartile ranges for continuous data and numbers and percentages for categorical data.

Mixed Poisson regression models with a random intercept per participant are used to analyse repeated count data (number of correct answers to questions for each case), mixed logistic regression for binary confidence variables and mixed linear regression models to analyze continuous repeated measurements like the NASA TLX scores for workload.

In addition to the modality (the actual covarite of interest), we include the case, age, gender and the center in the models. Note that work experience was not included in the models because the age and the experience were too closely related, causing multicollinearity.

3 Descriptives

In this section, we show some descriptives.

3.1 Participants

**Variable**	**n**	**Min**	q_1	$\widetilde{x}$	$\bar{x}$	q_3	**Max**	**s**	**IQR**	**#NA**
Age	50	27	33.0	37.0	37.9	43.8	56	7.0	10.8	0
Experience	50	4	7.2	10.5	13.2	16.8	37	7.6	9.5	0

Table 1: Descriptive table per participant - continuous data

**Variable**	**Levels**	**n**	%	$\sum$%
Center	USZ	10	20.0	20.0
	Hirs	10	20.0	40.0
	Wurz	10	20.0	60.0
	Frank	10	20.0	80.0
	Barc	10	20.0	100.0
	all	50	100.0	
Gender	female	19	38.0	38.0
	male	31	62.0	100.0
	all	50	100.0	
Profession	phys	25	50.0	50.0
	nurse	25	50.0	100.0
	all	50	100.0	

Table 2: Descriptive table per participant - categorical data

3.2 Solutions to the questions

Variable	Levels	n	Min	q_1	$\tilde{x}$	$\bar{x}$	q_3	Max	s	IQR	#NA
NumberCorrect	Conv	250	2	9	11	11.3	13	20	3.4	4	0
	VP	250	1	12	14	14.1	16	21	3.3	4	0
	all	500	1	10	13	12.7	15	21	3.7	5	0
Total	Conv	250	22	22	22	22.0	22	22	0.0	0	0
	VP	250	22	22	22	22.0	22	22	0.0	0	0
	all	500	22	22	22	22.0	22	22	0.0	0	0

Table 3: Number of correct decisions per modality

		Answer											
		low	safe	high	not m	no rec	asl	aw	n ST	abn ST	rela	n rela	Total
Corr	low	464	160	26	24	126	0	0	0	0	0	0	800
	safe	217	1592	186	94	511	0	0	0	0	0	0	2600
	high	74	155	767	31	173	0	0	0	0	0	0	1200
	not m	26	187	34	309	344	0	0	0	0	0	0	900
	asl	0	0	0	10	45	222	23	0	0	0	0	300
	aw	0	0	0	10	33	31	126	0	0	0	0	200
	n ST	0	0	0	0	85	0	0	197	18	0	0	300
	abn ST	0	0	0	0	63	0	0	50	87	0	0	200
	rela	0	0	0	0	76	0	0	0	0	89	35	200
	n rela	0	0	0	0	104	0	0	0	0	25	171	300
	Total	781	2094	1013	478	1560	253	149	247	105	114	206	7000

Table 4: Correct versus actual answers (diagnosis-related questions only)

		Answer<br>no rec	V.j.l	V.j.r	V.s.l	V.s.r	V.f.l	V.f.r	no	a l	a r	1 l	l r	A.f.r	A.f.l	tube	y	Total
Corr	V.j.l	21	55	8	5	1	5	0	5	0	0	0	0	0	0	0	0	100
	V.j.r	23	17	43	4	5	2	2	4	0	0	0	0	0	0	0	0	100
	V.s.l	20	26	15	27	8	0	0	4	0	0	0	0	0	0	0	0	100
	no	440	18	15	5	1	1	1	832	39	26	7	9	4	13	22	67	1500
	a l	126	0	0	0	0	0	0	35	261	62	12	4	0	0	0	0	500
	a r	35	0	0	0	0	0	0	2	20	40	1	2	0	0	0	0	100
	1 l	15	0	0	0	0	0	0	7	13	3	54	8	0	0	0	0	100
	A.f.r	23	0	0	0	0	0	0	22	0	0	0	0	42	13	0	0	100
	A.f.l	26	0	0	0	0	0	0	21	0	0	0	0	7	46	0	0	100
	tube	63	0	0	0	0	0	0	27	0	0	0	0	0	0	310	0	400
	y	174	0	0	0	0	0	0	97	0	0	0	0	0	0	0	629	900
	Total	966	116	81	41	15	8	3	1056	333	131	74	23	53	72	332	696	4000

Table 5: Correct versus actual answers (installation-related questions only)

3.3 Confidence and workload

Variable	Levels	n_{Conv}	$\%_{Conv}$	$\sum\%_{Conv}$	n_{VP}	$\%_{VP}$	$\sum\%_{VP}$	n_{all}	$\%_{all}$	$\sum\%_{all}$
Case	1	0	0.0	0.0	50	20.0	20.0	50	10.0	10.0
	2	0	0.0	0.0	50	20.0	40.0	50	10.0	20.0
	3	0	0.0	0.0	50	20.0	60.0	50	10.0	30.0
	4	0	0.0	0.0	50	20.0	80.0	50	10.0	40.0
	5	0	0.0	0.0	50	20.0	100.0	50	10.0	50.0
	6	50	20.0	20.0	0	0.0	100.0	50	10.0	60.0
	7	50	20.0	40.0	0	0.0	100.0	50	10.0	70.0
	8	50	20.0	60.0	0	0.0	100.0	50	10.0	80.0
	9	50	20.0	80.0	0	0.0	100.0	50	10.0	90.0
	10	50	20.0	100.0	0	0.0	100.0	50	10.0	100.0
	all	250	100.0		250	100.0		500	100.0	
Confidence	very unconf	20	8.0	8.0	2	0.8	0.8	22	4.4	4.4
	unconf	133	53.2	61.2	98	39.2	40.0	231	46.2	50.6
	conf	89	35.6	96.8	138	55.2	95.2	227	45.4	96.0
	very conf	8	3.2	100.0	12	4.8	100.0	20	4.0	100.0
	all	250	100.0		250	100.0		500	100.0	

Table 6: Cases and confidence by modality

Variable	Levels	n	Min	q_1	$\widetilde{x}$	$\bar{x}$	q_3	Max	s	IQR	#NA
Mental_NASA	Conv	250	10	57.5	70.0	69.0	82.0	100	19.3	24.5	0
	VP	250	6	47.2	61.5	60.4	75.0	100	20.3	27.8	0
	all	500	6	50.0	68.0	64.7	79.2	100	20.2	29.2	0
Temporal_NASA	Conv	250	16	67.0	76.5	75.4	87.0	100	18.0	20.0	0
	VP	250	4	52.2	67.0	65.4	79.8	100	19.8	27.5	0
	all	500	4	60.0	72.0	70.4	84.0	100	19.5	24.0	0
Performance_NASA	Conv	250	7	51.0	70.0	65.4	81.0	100	21.4	30.0	0
	VP	250	5	38.0	57.0	55.8	74.0	100	22.2	36.0	0
	all	500	5	43.0	65.0	60.6	77.0	100	22.3	34.0	0
Effort_NASA	Conv	250	7	62.0	75.0	72.2	85.0	100	19.4	23.0	0
	VP	250	4	50.0	67.5	64.1	78.0	100	20.2	28.0	0
	all	500	4	53.0	72.0	68.2	82.0	100	20.2	29.0	0
Frustration_NASA	Conv	250	8	52.2	70.0	65.9	79.0	100	18.8	26.8	0
	VP	250	8	45.0	59.0	57.2	71.8	100	18.5	26.8	0
	all	500	8	49.8	65.0	61.5	75.0	100	19.1	25.2	0
Overall_NASA	Conv	250	16	51.0	59.0	58.1	65.0	83	11.8	14.0	0
	VP	250	8	41.2	51.0	50.5	60.0	81	13.2	18.8	0
	all	500	8	46.0	55.0	54.3	63.0	83	13.1	17.0	0

Table 7: Workload by modality

3.4 General questions

Variable	Levels	n	%	$\sum$%
Qu_Overview	str dis	0	0.0	0.0
	dis	3	6.0	6.0
	neutral	11	22.0	28.0
	agree	28	56.0	84.0
	str agree	8	16.0	100.0
	all	50	100.0	
Qu_Use	str dis	1	2.0	2.0
	dis	10	20.4	22.4
	neutral	11	22.4	44.9
	agree	20	40.8	85.7
	str agree	7	14.3	100.0
	all	49	100.0	
Qu_Split	dis	7	14.0	14.0
	neutral	5	10.0	24.0
	agree	24	48.0	72.0
	str agree	14	28.0	100.0
	all	50	100.0	
Qu_myself	str dis	1	2.0	2.0
	dis	3	6.0	8.0
	neutral	16	32.0	40.0
	agree	17	34.0	74.0
	str agree	13	26.0	100.0
	all	50	100.0	

Table 8: General questions

4 Number of correct answers

We first perform McNemar's test for paired binary data to compare the number of correct decisions between the two modalities. Note, however, that this is not fully correct, because it assumes that there is independence between each of the pairs.

In our case we have repeated measurements, therefore the two modalities are compared using a mixed Poisson regression model with random intercept per individual while adjusting for potentially relevant covariates.

4.1 McNemar's test

		Conv<br>False	Correct	Total
VP	False	1153 (21.0%)	813 (14.8%)	1966 (35.7%)
	Correct	1518 (27.6%)	2016 (36.7%)	3534 (64.3%)
	Total	2671 (48.6%)	2829 (51.4%)	5500 (100.0%)

Table 9: Pairs of decisions (same case and question)

Table 9 shows the pairs of answers of the same participant for the same case and question, using the two different modalities. This table is the basis of McNemar's test.

McNemar's test results in a p-value of < 0.0001, which means that there is very strong evidence that there is a difference between the two modalities.

4.2 Mixed Poisson regression

Note that we changed the case number to 1 to 5 from now on, so that the same cases (who differ only in the modality, and this is already contained in the modality variable) are recognized and can be included in the models.

In the mixed Poisson regression model a random intercept for each participant is used, and we additionally adjust for age, gender and work experience.

	Rate ratio	CI lower	CI upper	p-value
Intercept	10.71	7.459	15.388	< 0.0001
Modality: VP	1.25	1.189	1.312	< 0.0001
Case 2	0.98	0.902	1.055	0.53
Case 3	1.08	1	1.166	0.05
Case 4	0.92	0.847	0.994	0.03
Case 5	1.06	0.983	1.146	0.13
Age	1	0.993	1.009	0.76
Sex: male	0.96	0.868	1.06	0.41
Center: Hirs	1.06	0.914	1.228	0.44
Center: Wurz	1.08	0.924	1.269	0.33
Center: Frank	1.06	0.899	1.242	0.50
Center: Barc	1.09	0.927	1.289	0.29
Job: Nurse	0.92	0.839	1.015	0.10

Table 10: Results for mixed Poisson regression model for number of correct answers per case

The results in Table 10 show that there is very strong evidence for a difference between the two modalities: The rate of correct decisions is about 1.25 times as high using visual patient in comparison to the conventional monitor.

There also is some weak to moderate evidence that the case 3 is slightly easier to solve and that case 4 is a bit more difficult than case 1. No evidence for an influence of the remaining variables can be seen.

Table 11: Matched odds ratios for the different questions (descriptive)

Question	OR	Conf.Lower	Conf.Upper
18	6.12	3.66	10.22
21	5.33	3.22	8.82
14	4.43	2.76	7.11
15	4.17	2.65	6.58
13	3.92	2.50	6.13
17	3.77	2.45	5.81
20	3.67	2.40	5.61
16	3.32	2.18	5.07
19	3.16	1.89	5.29
11	2.64	1.71	4.08
6	1.91	1.27	2.88
22	1.86	1.24	2.79
3	1.82	1.23	2.69
7	1.70	1.08	2.67
8	1.41	0.98	2.03
12	1.20	0.82	1.75
10	1.19	0.81	1.76
4	1.07	0.74	1.56
9	0.75	0.50	1.14
1	0.75	0.43	1.32
5	0.46	0.31	0.68
2	0.17	0.10	0.30

4.3 Odds ratios per modality

To explore the matter further, we calculated crude matched odds ratios as a descriptive measure (i.e. without any testing or p-values involved) to compare the different questions. In Table 11, where the questions are ordered according to the size of the odds ratios, we can see that for most questions, VP seems to be clearly better (as indicated by an odds ratio above 1), whereas for a few questions the conventional screen seems to be easier.

4.4 Separate models for diagnoses and installations

In this section, we calculate two mixed Poisson models as done previously, but separately for the diagnosis-related questions (questions 1 to 14) and for the installation-related questions (questions 15 to 22).

	Rate ratio	CI lower	CI upper	p-value
Intercept	8.3	5.598	12.311	< 0.0001
Modality: VP	1.1	1.031	1.167	0.0033
Case 2	0.97	0.879	1.067	0.52
Case 3	1.03	0.936	1.134	0.54
Case 4	0.89	0.809	0.987	0.03
Case 5	0.99	0.895	1.086	0.77
Age	1	0.991	1.008	0.88
Sex: male	0.97	0.871	1.081	0.59
Center: Hirs	1.02	0.871	1.199	0.79
Center: Wurz	1.02	0.859	1.214	0.81
Center: Frank	1.05	0.881	1.249	0.59
Center: Barc	1.07	0.895	1.28	0.46
Job: Nurse	0.9	0.812	0.997	0.04

Table 12: Results for mixed Poisson regression model for number of correct answers per case; diagnosis-related questions only

In the model for the diagnosis, there is strong evidence for an effect of the modality with a rate ratio of 1.1, and moderate evidence for the job description: Compared to a physician, the rate of correct diagnoses for a nurse is only 0.9 times as high. In additiona, moderate evidence for a difference between case 4 and 1 can be seen, but no evidence for an effect of the remaining variables.

In the model for the installations there is even very strong evidence for an effect (and the estimated effect is stronger) with a rate ratio of 1.57. Strong evidence for differences between the cases 3 and 5 in comparison to case 1 can be seen, but apart from that there is no evidence for an effect of the other covariates.

5 Confidence

In this section, we use mixed logistic regression models to quantify differences between the two modalities with respect to the question if the participant felt confident with his decision after each case.

Note that again, the cases 6 to 10 get the same numbers 1 to 5 as in the former chapter. We created a binary confidence variable, summarizing the values ”very confident” and ”confident” to one value and in the same way the values ”unconfident” and ”very unconfident” to one value, so that the regression model is more easily understandable.

The results of the mixed logistic regression model for binary confidence can be seen in Table

	Rate ratio	CI lower	CI upper	p-value
Intercept	2.71	1.761	4.165	< 0.0001
Modality: VP	1.57	1.445	1.706	< 0.0001
Case 2	0.99	0.866	1.129	0.86
Case 3	1.17	1.033	1.332	0.01
Case 4	0.96	0.843	1.101	0.59
Case 5	1.2	1.06	1.365	0.0042
Age	1	0.996	1.014	0.29
Sex: male	0.94	0.837	1.057	0.30
Center: Hirs	1.12	0.945	1.336	0.19
Center: Wurz	1.19	0.988	1.43	0.07
Center: Frank	1.07	0.884	1.293	0.49
Center: Barc	1.13	0.93	1.368	0.22
Job: Nurse	0.96	0.856	1.069	0.43

Table 13: Results for mixed Poisson regression model for number of correct answers per case; installation-related questions only

14. There is very strong evidence for a difference between the two modalities: With visual patient, the odds for being confident were about 3.32 times as high than with the conventional monitor. Weak evidence for a difference between case 3 and case 1 can be seen. Apart from that there is no evidence for an effect of any of the other variables.

	Odds ratio	CI lower	CI upper	p-value
Intercept	0.13	0	3.69	0.23
Modality: VP	3.32	2.15	5.11	< 0.0001
Case 2	1.03	0.53	1.99	0.93
Case 3	1.83	0.94	3.55	0.07
Case 4	1.22	0.64	2.36	0.54
Case 5	0.97	0.51	1.88	0.94
Age	1.01	0.94	1.09	0.72
Sex: male	2.41	0.97	5.98	0.06
Center: Hirs	1.5	0.38	5.97	0.56
Center: Wurz	1.12	0.25	4.9	0.88
Center: Frank	1.41	0.31	6.32	0.65
Center: Barc	1.35	0.29	6.33	0.70
Job: Nurse	0.96	0.4	2.32	0.94

Table 14: Results for mixed logistic regression model for binary confidence

6 Analysis of workload

Workload was measured with the NASA TLX score. We use a linear mixed model to analyse the overall NASA TLX score as main summary measure for workload, but we don't fit any models for its subscales to avoid multiple testing. For a descriptive comparison of the different subscores please see Table 7.

The model again used a random intercept for each participant and was adjusted for the same variables as the models above.

	Coefficient	CI lower	CI upper	p-value
Intercept	69.03	50.42	87.27	< 0.0001
Modality: VP	-7.62	-9.17	-6.07	< 0.0001
Case 2	1.51	-0.95	3.97	0.23
Case 3	-0.89	-3.35	1.57	0.48
Case 4	-1.38	-3.84	1.07	0.27
Case 5	0.51	-1.95	2.97	0.69
Age	-0.29	-0.68	0.12	0.19
Sex: male	-0.74	-5.86	4.2	0.79
Center: Hirs	2.38	-5.25	10.06	0.57
Center: Wurz	0.36	-7.78	8.61	0.94
Center: Frank	-4.12	-12.36	4.26	0.37
Center: Barc	-6.81	-15.29	1.77	0.15
Job: Nurse	4.39	-0.55	9.29	0.11

Table 15: Results for mixed linear regression model 1 for NASA TLX

The linear mixed model in Table 15 shows strong evidence that the VP leads to a workload that was on average -7.62 points lower compared to the conventional monitor. No evidence for an effect of the remaining variables could be seen.

R version and packages used to generate this report

R version: R version 4.0.5 (2021-03-31)

Base packages: stats, graphics, grDevices, utils, datasets, methods, base

Other packages: lmerTest, lme4, Matrix, tidyr, readr, dplyr, stringr, reporttools, xtable, ggplot2, knitr

This document was generated on 2021-11-04 at 17:27.
